# Multi-robot collision avoidance method in sweet potato fields

**DOI:** 10.3389/fpls.2024.1393541

**Published:** 2024-09-10

**Authors:** Kang Xu, Jiejie Xing, Wenbin Sun, Peng Xu, Ranbing Yang

**Affiliations:** ^1^ College of Information and Communication Engineering, Hainan University, Haikou, China; ^2^ College of Mechanical and Electrical Engineering, Hainan University, Haikou, China

**Keywords:** multi-robot, accurate spraying, collision avoidance, sweet potatoes, itinerary table

## Abstract

Currently, precise spraying of sweet potatoes is mainly accomplished through semi-mechanized or single spraying robots, which results in low operating efficiency. Moreover, it is time-consuming and labor-intensive, and the pests and diseases cannot be eliminated in time. Based on multi robot navigation technology, multiple robots can work simultaneously, improving work efficiency. One of the main challenges faced by multi robot navigation technology is to develop a safe and robust collision avoidance strategy, so that each robot can safely and efficiently navigate from its starting position to the expected target. In this article, we propose a low-cost multi-robot collision avoidance method to solve the problem that multiple robots are prone to collision when working in field at the same time. This method has achieved good results in simulation. In particular, our collision avoidance method predicts the possibility of collision based on the robot’s position and environmental information, and changes the robot’s path in advance, instead of waiting for the robot to make a collision avoidance decision when it is closer. Finally, we demonstrate that a multi-robot collision avoidance approach provides an excellent solution for safe and effective autonomous navigation of a single robot working in complex sweet potato fields. Our collision avoidance method allows the robot to move forward effectively in the field without getting stuck. More importantly, this method does not require expensive hardware and computing power, nor does it require tedious parameter tuning.

## 1 Introduction

Sweet potato is an important food crop after rice, wheat, and corn. With the development of large-scale sweet potato cultivation, sweet potato diseases and pests are becoming increasingly serious. Traditional prevention and treatment methods are high in cost and low in efficiency, and there are problems such as prevention and treatment of chemical abuse and overuse. Harmful pesticide chemicals will spread into the air, causing pollution to the environment and sweet potato crops, and may enter farmers’ bodies through the respiratory system (Meshram et al., 2022). Precision spraying technology can solve the problems of pesticide waste and overuse, improve pesticide utilization, and reduce pesticide pollution to the environment (Xiongkui, 2020). However, in the current precision spraying operations, a single robot is mainly used for spraying operations, such as Danton et al., 2020; Baltazar et al., 2021; Oberti and Schmilovitch, 2021, and Liu et al., 2022. A single robot has the disadvantages of limited operating capacity and long operating time. Especially for sweet potato fields with a relatively large area, using a single robot for spraying will take a lot of time (Kim and Son, 2020), and pests and diseases cannot be eliminated in time, causing irreparable economic losses to agriculture. In addition, if the robot fails, the entire spraying operation will be delayed, resulting in greater economic losses. Therefore, applying a multi-robot system to sweet potato spraying can effectively solve the problem of low operating efficiency of a single device, reduce agricultural maintenance costs, and indirectly improve agricultural economic benefits. There are many benefits to using a multi-robot system instead of a single robot (Parker et al., 2016; Jawhar et al., 2018): (1) Multiple robots can perform tasks simultaneously to complete tasks faster. (2) Multiple robots can effectively handle tasks that are essentially distributed over a wide area. (3) When any robot fails, multiple robots that can perform similar processes can be used to compensate.

As the number of agricultural workers continues to decrease around the world, the use of multi-robot systems to perform agricultural tasks has become increasingly common in large-scale fields with fewer people (Carbone et al., 2018), and multi-robot systems have gradually become a hot topic of research. However, one of the main challenges facing multi-robot systems is to develop a safe and robust collision avoidance strategy that enables each robot to navigate safely and efficiently from the starting position to the desired target (Long et al., 2017). The robot’s obstacle avoidance method can be divided into local obstacle avoidance and global path according to the operation requirements (Tang et al., 2024), such as Genetic algorithm (Zhao et al., 2021), dynamic window approach (Chang et al., 2021), RRT* (Hu et al., 2024), A* (Zhang et al., 2024) and other algorithms. However, these obstacle avoidance methods are all for a single robot and are not suitable for multi-robot systems (Cai et al., 2007). At present, the collision avoidance methods of multi-robots are mainly divided into centralized control and decentralized control.

Initially, scholars used a centralized control method to avoid collisions between multiple robots. This approach assumed that the behavior of all robots was determined by a central controller that had comprehensive knowledge of all robots’ intentions (e.g., initial states and goals) and their workspaces (e.g., 2D grid maps). Schwartz and Sharir (1983) proposed to find the continuous motion of two given structures connecting objects within a given region, during which they avoided collisions with walls and each other. He et al. (2016) used the cloud platform to calculate local collision-free paths for each robot, which improved the processing capabilities of the device. However, this method needed to ensure that each robot had networking capabilities. Yu and LaValle (2016); Tang et al. (2018); Matoui et al. (2020) and Cheng et al. (2023) proposed a centralized trajectory generation algorithm to find trajectories that navigate robots from starting positions to non-interchangeable target positions in a collision-free manner by planning optimal paths for all robots. In addition, Keshmiri and Payandeh (2009) used a centralized method to keep multiple robots in formation to avoid collisions between robots, but it was not suitable for the agricultural field and cannot control multiple robots to maintain formation during precise spraying. Centralized approaches can ensure multi-robot system safety, integrity, and near-optimality, but they are difficult to scale to large systems with many robots due to the high computational cost of multi-robot scheduling, heavy reliance on reliable synchronous communication, and low tolerance for failures or interference.

Compared with centralized methods, some existing studies proposed decentralized collision avoidance strategies, where each robot made decisions independently by considering the observable states (such as shape, speed, and position) of other robots. Cai et al. (2007) developed an advanced method for collision avoidance in multi-robot systems based on established techniques of omnidirectional vision systems, automatic control, and dynamic programming. However, each robot in this system must be an autonomous robot, equipped with equipment or systems such as omnidirectional vision systems, target recognition systems, communication systems, and control systems. Claes et al. (2012); Hennes et al. (2012), and Godoy et al. (2016) designed inter-agent communication protocols to share position and velocity information among nearby agents. However, communication systems posed additional difficulties, such as delays or blocking of communication signals due to obstruction by obstacles. Althoff et al. (2012) proposed a probabilistic threat assessment method for reasoning about the safety of robot trajectories. In this method, the trajectories of other dynamic obstacles needed to be sampled and then the global collision probability was calculated. Lacked of possibilities for safe navigation in dynamic multi-robot environments. Sun et al. (2014) proposed a behavior-based multi-robot collision avoidance method in large robot teams inspired by the concept of swarm intelligence, which could solve the coordination problem of multi-robots by using group behavior. Fiorini and Shiller (1998), Van den Berg et al. (2008); Snape et al. (2009); Hennes et al. (2012); Alonso-Mora et al. (2013); Claes and Tuyls (2018), and Zhu et al. (2020) designed a robot collision avoidance strategy based on the speed obstacle framework. This algorithm effectively inferred the local collision-free motion of multiple agents in a chaotic workspace. However, these methods have several serious limitations that hinder their widespread application in practical scenarios. First of all, it is difficult to meet the requirement that each agent has perfect perception of the surrounding environment. This requirement does not hold true in real scenarios. A second limitation is that speed barrier-based strategies are controlled by multiple adjustable parameters that are sensitive to scene settings and therefore must be set carefully to achieve satisfactory multi-robot locomotion.

Inspired by methods based on the speed obstacle framework, Chen et al. (2017b), and Chen et al. (2017a) trained a collision avoidance strategy using deep reinforcement learning. This strategy explicitly mapped the agent’s own state and that of its neighbors to collision-free actions, but it still required perfect awareness of the surrounding environment. Long et al. (2017); Pfeiffer et al. (2017); Wang et al. (2020); Huang et al. (2022); Xiao et al. (2022), and Han et al. (2022) used deep neural network modeling and trained using supervised learning on large datasets. However, there are several limitations to supervised learning policies. First, it requires a large amount of training data, which should cover different interaction situations of multiple robots, and the training time cost is high. Secondly, the expert trajectories in the data set are not guaranteed to be optimal in interactive scenarios, which makes it difficult for training to converge to a robust solution. Third, it is difficult to manually design a suitable loss function to train a robust collision avoidance strategy. Fourth, the hardware requirements required for model deployment on robots are relatively high. When the number of robots increases, the hardware cost increases exponentially. Multi-robot systems based on reinforcement learning or transfer learning require a large amount of data sets for training, which requires relatively high time and economic costs. However, the economic benefits obtained in agriculture are obviously not suitable for the large-scale application of such robots.

Ünal et al. (2023) proposed a robot collision avoidance algorithm for traveling along tangent paths to solve the problem of multi-robot collision avoidance in precision agriculture. However, they only considered two robots and did not introduce the specific application environment. In addition, they did not consider whether the robot would damage the surrounding crops when avoiding collision. Currently, research on multi-robot collision avoidance strategies mainly focuses on confined space scenes or in relatively open and empty spaces, and no scholars have considered how to avoid collisions among multi-robots in large field (Raibail et al., 2022). Due to the special field environment, the robot can only move along the ridges, and can only move forward or backward. It cannot move freely in all directions, otherwise it will damage the crops and the robot, causing unnecessary economic losses. The current multi-robot collision avoidance strategy cannot be directly applied to this situation. In order to overcome the shortcomings of the above method, we propose a low-cost multi-robot collision avoidance method, which uses position and known environmental information to predict the possibility of collision, and changes the robot path in advance to achieve the purpose of robot collision avoidance. The method outperforms existing agent- and sensor-level methods in terms of navigation speed and safety. It can be directly and smoothly deployed on physical robots, without the need for expensive laser radars, cameras, and terminal controllers, and without the need for cumbersome parameter adjustments. Based on this, we propose a multi-robot collision-free method suitable for the special environment of field. The main innovations and contributions are as follows:

1. The multi-robot collision avoidance method we proposed only requires the robot to have a communication module, a positioning module and a low-cost control chip. It does not require the robot to have vision, radar and other sensors, nor does it require the ability to deploy reinforcement learning models. Reduce farmers’ purchase costs and field maintenance costs, thereby increasing farmers’ economic income.
2. We proposed a rapid robot path generation method suitable for farmland. The global information of the farmland was generated through the two endpoints of the crop rows and the row spacing. The global information included the straight-line equation and the two endpoints of the crop rows, occupying very little storage space. Then based on the requirements of farm operations and the robot’s target location, we could quickly generate a global path for the robot.
3. We proposed a robot collision avoidance method that could determine its own direction and working status only based on global information and the robot’s position. Then the robot’s position and global information were used to predict the possibility of collision in advance. Finally, the robot’s local path was adjusted according to the robot’s priority to avoid collisions. This method could quickly find collision-free paths for multi-robot systems and can be safely generalized to other work scenarios.

The rest of this paper is as follows: Section 2 introduces the working environment of multi-robot systems, related definitions and collision types. In Section 3, we discussed in detail how to predict the possibility of robot collision through maps and robot positions, and how to adjust local paths in the special environment of field to avoid collisions among multiple robots. The fourth section introduces the simulation environment and simulation settings of the multi-robot collision avoidance method, and verifies the method proposed in the third section. The final section presents the summary and prospects of this study.

## 2 Materials and methods

### 2.1 The working environment of robots

**Figure 1:**
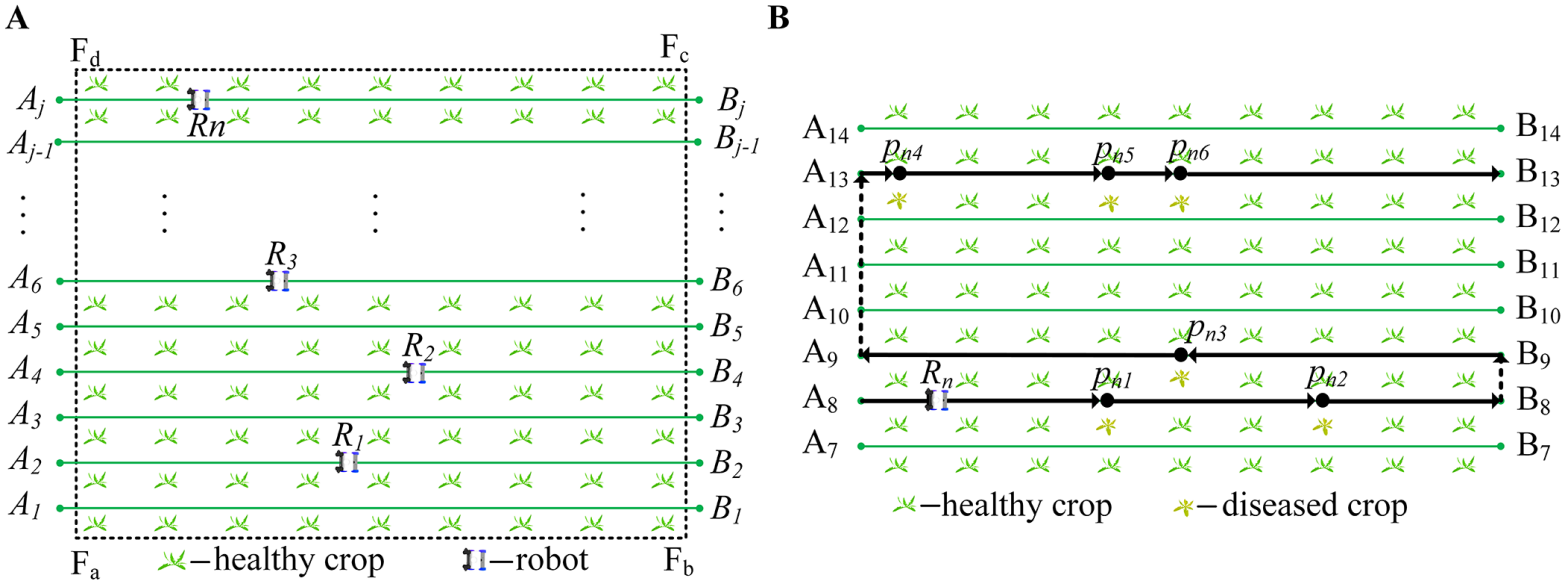
A simplified diagram of the robot’s working environment. **(A)** Simplified diagram of field. **(B)** The working path of the robot.

The sweet potato field is simplified to facilitate the description of the robot’s working conditions, as shown in **Figure 1**. The rectangle ${\text{F}}_{\text{a}}{\text{F}}_{\text{b}}{\text{F}}_{\text{c}}{\text{F}}_{\text{d}}$ represents the field, and $R_{n}\;\left(n\in N^{*}\right)$ represents the working robots (**Figure 1A**). The line segments $A_{j}B_{j}\;\left(j\in N^{*}\right)$ , $A_{s}A_{u}$ , and $B_{s}B_{u}\;(s\neq u,\;0<s\leq j,0<u\leq j)$ represent the robot’s paths in the field (**Figure 1B**). The line segment $A_{j}B_{j}$ is called the working path, the points $A_{j}$ and $B_{j}$ are the endpoints of the $A_{j}B_{j}$ , respectively, and j is the serial number of the working path. Line segments $A_{s}A_{u}$ and $B_{s}B_{u}$ are called the transition path. We set the sweet potato field to be planted in a standardized manner, with working paths running parallel to each other. The distance between each working path is represented by D .

#### 2.1.1 Definitions of robots

R  represents a group of working robots, as shown in Equation 1. $R_{n}$ has the same dynamic model in vertical and horizontal dimensions. The real-time location of $R_{n}$ is represented by $P_{R_{n}}$ , and $P_{R_{n}}$ is represented by coordinates $\left(x_{R_{n}},y_{R_{n}}\right)$ .


(1) $$\text{R}=\left\{{\text{R}}_{\text{n}}|\text{n}\in {\text{N}}^{*}\right\}$$

$R_{O}$ represents other robots except for $R_{n}$ , as shown in Equation 2.


(2) $${\text{R}}_{\text{O}}=\text{R}-{\text{R}}_{\text{n}}$$

A group of target points of robot $R_{n}$ is represented by $P_{n}$ , as shown in Equation 3. Robot $R_{n}$ moves at a constant speed along the target point. After robot $R_{n}$ reaches target $P_{n}$ , it stops for 3 seconds and performs precision spraying operations.


(3) $${\text{P}}_{\text{n}}=\left\{{\text{p}}_{\text{ni}}|{\text{p}}_{\text{ni}}\;\in \;{\text{R}}^{2},\text{n}\;\in \;{\text{N}}^{*},\text{i}\;\in \;{\text{N}}^{*}\right\}$$

where the coordinates of $p_{ni}$ are represented by $\left(x_{ni},y_{ni}\right)$ , and $x_{ni}$ and $y_{ni}$ are the horizontal and vertical coordinates on a two-dimensional plane, respectively. $r^{n}$ represents the global path of the robot, and $r_{j}^{n}$ represents the local path of the robot, as shown in Equation 4. The local path comprises a series of points, such as $r_{8}^{n}=\left\{A_{8},p_{n1},p_{n2},B_{8}\right\}$ and $r_{9}^{n}=\left\{B_{9},p_{n3},A_{9}\right\}$ , as shown in the solid black line in **Figure 1B**. $r^{n}$ comprises a series of local paths, such as all-black paths in **Figure 1B**.


(4) $$r^{n}=\{r_{j}^{n}|\text{n}\;\in \;{\text{N}}^{*},\;\text{j}\;\in \;{\text{N}}^{*}\}$$

There are two types of paths: the working and transition. In **Figure 1B**, the solid black lines $A_{8}B_{8}$ , $A_{9}B_{9}$ , and $A_{13}B_{13}$ are the working path, and the dotted black lines $B_{8}B_{9}$ and $A_{9}A_{13}$ are the transition path.

The robot has different moving directions on the working and transition paths. When the robot is moving on the working path, the robot’s direction at points $A_{j}$ and $B_{j}$ are 0 and 1, respectively. When going from point $A_{j}$ to $B_{j}$ , the direction of the robot is 0→1 . When the robot goes from point $B_{j}$ to $A_{j}$ , the robot’s direction is 1→0 . Therefore, we can get the direction of the robot only through the two endpoints of the local path without using other sensors. The expression method of the robot’s moving direction in the working path is shown in Equation 5.


(5) $$\begin{array}{l} {\text{r}}_{8}^{\text{n}}=\left\{{\text{A}}_{8},{\text{p}}_{\text{n}1},{\text{p}}_{\text{n}2},{\text{B}}_{8}\right\}\left(0\to 1\right)\\ {\text{r}}_{9}^{\text{n}}=\left\{{\text{B}}_{9},{\text{p}}_{\text{n}3},{\text{A}}_{9}\right\}\left(1\to 0\right) \end{array}$$

When the robot moves on the transition path, the direction is determined according to the serial number of the current and last working paths, as shown in Equation 6. The robot’s direction is up when the sign is a positive number. When the sign is negative, the direction of the robot is down . The direction on the transition path is only judged by the j , and no other sensors are needed.


(6) $$\text{sign}={\text{j}}_{\text{c}}-{\text{j}}_{\text{l}}$$

where $j_{c}$ represents the serial number of the current working path, and $j_{l}$ represents the serial number of the last working path.

#### 2.1.2 The working rules of robots

The width of the working path $A_{j}B_{j}$ in the field is relatively narrow, and it does not support operations such as moving side by side or turning; otherwise, the robots will crush or knock down the crops. Therefore, robots with different moving directions cannot simultaneously exist in the working path $A_{j}B_{j}$ ; otherwise, the robots will collide.

The robots move in a ‘U’ shape in the field (Hameed et al., 2013), and the working method is shown in the black path in **Figure 1B**. For example, $R_{n}$ starts from point $A_{8}$ , moves along the working path $A_{8}B_{8}$ , reaches the end point $B_{8}$ , and moves along $B_{8}B_{9}$ to $A_{9}B_{9}$ . In addition, robots start from the garage, go to the field to work, and then return to the garage.

### 2.2 Collision and conflict scenarios of robots

During the research, we found that the possibility of a robot collision can be judged based on the robot’s path type. When robots move on the same path type, we can determine whether the robots will collide by simply comparing their serial number of the working paths and directions. The path type and serial number are the same, indicating that they are in the same working path. At this time, if they are moving in opposite directions, it means that they are moving toward each other and a collision will inevitably occur. If they are moving in the same direction, as long as their speeds are the same, there will be no collision. Therefore, we divide the robot collision scenarios into four types based on the different positions of $R_{1}$ and $R_{2}$ : 1) working path and different movement directions; 2) working path and same movement direction; 3) transition path and same movement direction; 4) transition path and different movement directions.

**Figure 2:**
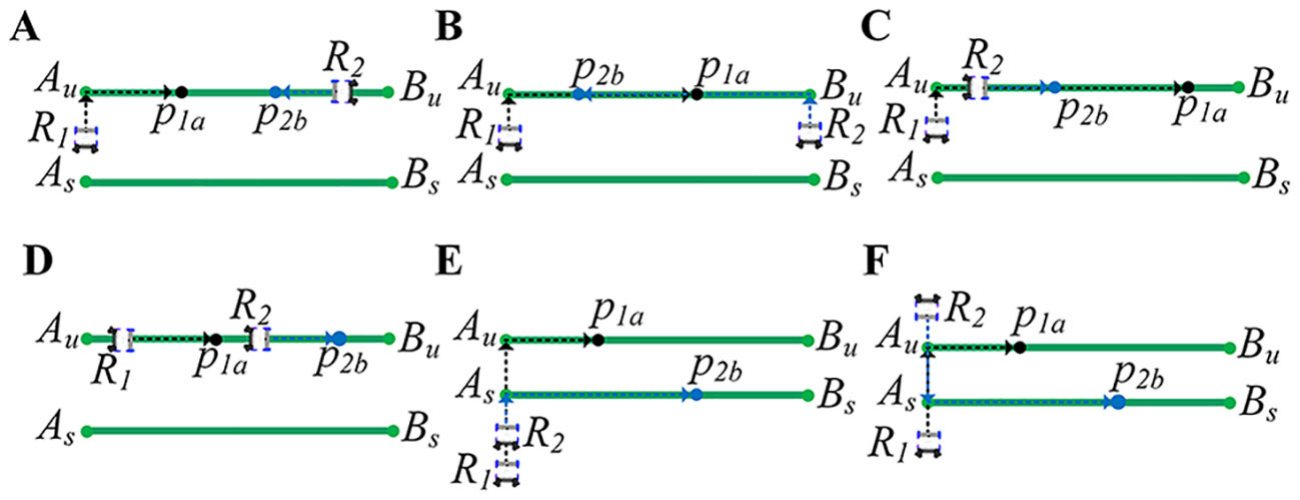
Types of robot conflicts. **(A, B)** Working path and different movement directions. **(C, D)** Working path and same movement direction. **(E)** Transition path and same movement direction. **(F)** Transition path and different movement directions.

As shown in **Figure 2**, it describes the possibility of collision when the robot is working. The green line segments represent the working paths, and the two working robots are represented by $R_{1}$ and $R_{2}$ ; their target points are $p_{1a}$ and $p_{2b}$ .

#### 2.2.1 Working path and different movement directions

**Figure 2A** describes that robot $R_{2}$ has been moving in the working path $A_{u}B_{u}$ , and robot $R_{1}$ is about to enter the working path $A_{u}B_{u}$ . Robots $R_{1}$ and $R_{2}$ move in opposite directions. According to section 2.1, there cannot be two robots with different moving directions in the same working path. If robot $R_{1}$ continues to enter the working path $A_{u}B_{u}$ without changing its path, it will inevitably collide with $R_{2}$ .

**Figure 2B** describes that $R_{1}$ and $R_{2}$ are about to enter the same working path $A_{u}B_{u}$ . Robots $R_{1}$ and $R_{2}$ are moving in opposite directions. If the path of robot $R_{1}$ or $R_{2}$ is not changed, $R_{1}$ and $R_{2}$ will eventually collide together.

#### 2.2.2 Working path and same movement direction

**Figure 2C** describes that robot $R_{2}$ is already moving in the working path $A_{u}B_{u}$ , and robot $R_{1}$ is about to enter the working path $A_{u}B_{u}$ . **Figure 2D** describes that the robots $R_{1}$ and $R_{2}$ are already moving in the working path $A_{u}B_{u}$ . Robots $R_{1}$ and $R_{2}$ move in the same direction. According to Section 2.1, two arobots with the same moving direction can exist in the same working path. Since the speeds of the robots are the same, there will be a constant distance between robots moving in the same direction. However, when robot $R_{2}$ arrives at target point $p_{2b}$ , the spraying operation takes time, and robot $R_{1}$ is still moving normally. $R_{1}$ needs to keep a safe distance from $R_{2}$ to stop moving and then wait for $R_{2}$ to complete its work. Otherwise, $R_{1}$ and $R_{2}$ will collide together.

#### 2.2.3 Transition path and same movement direction

**Figure 2E** describes that $R_{1}$ and $R_{2}$ move in the same direction and go to different working paths. Since $R_{2}$ and $R_{1}$ move in the same direction and speed, they will not collide. However, when $R_{2}$ goes to the working path $A_{s}B_{s}$ where the target point $p_{2b}$ is located, it will turn around, which takes a certain amount of time. If $R_{1}$ does not stop moving and waits for $R_{2}$ to complete its turn, $R_{1}$ and $R_{2}$ will collide.

#### 2.2.4 Transition path and different movement directions

**Figure 2F** describes that $R_{1}$ and $R_{2}$ move in different directions and go to different working paths. When $R_{1}$ goes to the target point $p_{1a}$ , $R_{2}$ goes to the target point $p_{2b}$ from the opposite direction. If the path of $R_{1}$ or $R_{2}$ is not changed, they will collide at a particular moment.

## 3 Approach

### 3.1 Global map generation method

In order to get the serial number j of working path and the robot’s moving direction, the geographic information is needed. Use straight lines $A_{j}B_{j}$ and the endpoints $A_{j}$ and $B_{j}$ to generate a field map. Represent this map as a point-line map ( plm ). Use straight line $A_{1}B_{1}$ as the baseline to generate other parallel line segment. As described in **Algorithm 1**, according to the straight-line equation of $A_{1}B_{1}$ and the distance D between each working path, the straight-line equations of all $A_{j}B_{j}$ and the coordinates of the endpoints $A_{j}$ and $B_{j}$ are obtained. Finally, all working paths $A_{j}B_{j}$ and endpoints $A_{j}$ and $B_{j}$ are stored in the plm .

Algorithm 1. The plm generation method 
Input: the coordinates 
$\left(x_{A_{1}},y_{A_{1}}\right)$
, and 
$\left(x_{B_{1}},y_{B_{1}}\right)$
 of the two endpoints
$\;A_{1}$
 and 
$B_{1}$


Output: 
plm


1: Initialize 
$k_{A_{1}B_{1}}$
 and 
$b_{A_{1}B_{1}}$


2: **for** *j* = 2, 3,…, 
$N^{*}$
 **do**

3:   
$k_{A_{j}B_{j}}$
 
=
 
$k_{A_{1}B_{1}}$


4:   
$b_{A_{j}B_{j}}$
 
=
 
$b_{A_{1}B_{1}}+\left(j-1\right)D\sqrt{k_{A_{1}B_{1}}^{2}+1}$


5:   //Update straight-line equation of 
$A_{j}B_{j}$


6:   
$y_{A_{j}B_{j}}$
 
=
 
$k_{A_{j}B_{j}}x_{A_{j}B_{j}}+b_{A_{j}B_{j}}$


7:   //Update straight-line equations of 
$A_{1}A_{j}$
 and 
$B_{1}B_{j}$


8:   
$k_{A_{1}A_{j}}$
, 
$k_{B_{1}B_{j}}$
 
=
 
$-1/k_{A_{1}B_{1}}$


9:   
$y_{A_{1}A_{j}}$
, $y_{B_{1}B_{j}}$
  =
 -  $x_{A_{1}A_{j}}/k_{A_{j}B_{j}}+b_{A_{1}A_{j}}$
, -  $x_{B_{1}B_{j}}/k_{A_{j}B_{j}}+b_{B_{1}B_{j}}$


10:   $b_{A_{1}A_{j}}$
,  $b_{B_{1}B_{j}}$
  =
  $y_{A_{1}A_{j}}+x_{A_{1}A_{j}}/k_{A_{j}B_{j}}$
,  $y_{B_{1}B_{j}}+x_{B_{1}B_{j}}/k_{A_{j}B_{j}}$


11:  //Update coordinates of  $A_{j}$
 and  $B_{j}$


12:   $A_{j}(x_{A_{j}},y_{A_{j}})\;$


←
 (
$b_{A_{1}A_{j}}-b_{A_{j}B_{j}}$
)
$/\left(k_{A_{j}B_{j}}-k_{A_{1}A_{j}}\right)$
,
$k_{A_{1}A_{j}}x_{A_{j}}+b_{A_{1}A_{j}}$


13:   $B_{j}(x_{B_{j}},y_{B_{j}})\;$


← 


$\left(b_{B_{1}B_{j}}-b_{A_{j}B_{j}}\right)/\left(k_{A_{j}B_{j}}-k_{B_{1}B_{j}}\right)$
,
$k_{B_{1}B_{j}}x_{B_{j}}+b_{B_{1}B_{j}}$


14: **end for**

15: *plm*
← 
$\left\{A_{j}B_{j},A_{j},B_{j}|j\epsilon N^{*}\right\}$


16: **return** *plm*

#### 3.1.1 Calculating the equation of the working path

As shown in **Figure 1**, take $A_{1}B_{1}$ as the baseline, and make j parallel line segments $A_{1}B_{1}$ , $A_{2}B_{2}$ ,…,. Suppose the slope of line segment $A_{1}B_{1}$ is $k_{A_{1}B_{1}}$ , and the intercept is $b_{A_{1}B_{1}}$ , then the straight-line equation of line segment $A_{1}B_{1}$ is expressed as:


(7) $${\text{y}}_{{\text{A}}_{1}{\text{B}}_{1}}={\text{k}}_{{\text{A}}_{1}{\text{B}}_{1}}{\text{x}}_{{\text{A}}_{1}{\text{B}}_{1}}+{\text{b}}_{{\text{A}}_{1}{\text{B}}_{1}}$$

The coordinates $\left(x_{A_{1}},y_{A_{1}}\right)$ , and $\left(x_{B_{1}},y_{B_{1}}\right)$ of the two endpoints and $B_{1}$ of $A_{1}B_{1}$ are obtained through the positioning device. Then the slope $k_{A_{1}B_{1}}$ and intercept $b_{A_{1}B_{1}}$ of $A_{1}B_{1}$ are expressed as:


(8) $${\text{k}}_{{\text{A}}_{1}{\text{B}}_{1}}=\left({\text{y}}_{{\text{B}}_{1}}-{\text{y}}_{{\text{A}}_{1}}\right)/\left({\text{x}}_{{\text{B}}_{1}}-{\text{x}}_{{\text{A}}_{1}}\right)$$

(9) $${\text{b}}_{{\text{A}}_{1}{\text{B}}_{1}}={\text{y}}_{{\text{A}}_{1}}-{\text{k}}_{{\text{A}}_{1}{\text{B}}_{1}}{\text{x}}_{{\text{A}}_{1}}$$

Since the straight line $A_{1}B_{1}$ is parallel to the straight line $A_{2}B_{2}$ , the slope $k_{A_{1}B_{1}}$ of the straight line $A_{1}B_{1}$ is equal to the slope $k_{A_{2}B_{2}}$ of the straight line $A_{2}B_{2}$ . According to the distance formula between two parallel lines, the intercept $b_{A_{2}B_{2}}$ of the straight line $A_{2}B_{2}$ is expressed as:


(10) $${\text{b}}_{{\text{A}}_{2}{\text{B}}_{2}}={\text{b}}_{{\text{A}}_{1}{\text{B}}_{1}}+\text{D}\sqrt{{\text{k}}_{{\text{A}}_{1}{\text{B}}_{1}}^{2}+1},{\text{b}}_{{\text{A}}_{2}{\text{B}}_{2}}>{\text{b}}_{{\text{A}}_{1}{\text{B}}_{1}}$$

According to Equation 10, the straight-line equation of $A_{2}B_{2}$ is expressed as:


(11) $${\text{y}}_{{\text{A}}_{2}{\text{B}}_{2}}={\text{k}}_{{\text{A}}_{1}{\text{B}}_{1}}{\text{x}}_{{\text{A}}_{2}{\text{B}}_{2}}+{\text{b}}_{{\text{A}}_{1}{\text{B}}_{1}}+\text{D}\sqrt{{\text{k}}_{{\text{A}}_{1}{\text{B}}_{1}}^{2}+1}$$

The distance from straight-line segment $A_{3}B_{3}$ to straight-line segment $A_{1}B_{1}$ is 2 D . Then according to Equation 10, the intercept $b_{A_{3}B_{3}}$ of the straight line $A_{3}B_{3}$ is expressed as:


(12) $${\text{b}}_{{\text{A}}_{3}{\text{B}}_{3}}={\text{b}}_{{\text{A}}_{1}{\text{B}}_{1}}+2\text{D}\sqrt{{\text{k}}_{{\text{A}}_{1}{\text{B}}_{1}}^{2}+1},\;{\text{b}}_{{\text{A}}_{3}{\text{B}}_{3}}>{\text{b}}_{{\text{A}}_{1}{\text{B}}_{1}}$$

According to Equation 12, the straight-line equation of $A_{3}B_{3}$ is expressed as:


(13) $${\text{y}}_{{\text{A}}_{3}{\text{B}}_{3}}={\text{k}}_{{\text{A}}_{3}{\text{B}}_{3}}{\text{x}}_{{\text{A}}_{3}{\text{B}}_{3}}+{\text{b}}_{{\text{A}}_{3}{\text{B}}_{3}}={\text{k}}_{{\text{A}}_{1}{\text{B}}_{1}}{\text{x}}_{{\text{A}}_{3}{\text{B}}_{3}}+{\text{b}}_{{\text{A}}_{1}{\text{B}}_{1}}+2\text{D}\sqrt{{\text{k}}_{{\text{A}}_{1}{\text{B}}_{1}}^{2}+1}$$

According to Equations 10-13, the straight-line equation of $A_{j}B_{j}$ is expressed as:


(14) $${\text{y}}_{{\text{A}}_{\text{j}}{\text{B}}_{\text{j}}}={\text{k}}_{{\text{A}}_{1}{\text{B}}_{1}}{\text{x}}_{{\text{A}}_{\text{j}}{\text{B}}_{\text{j}}}+{\text{b}}_{{\text{A}}_{1}{\text{B}}_{1}}+\left(\text{j}-1\right)\text{D}\sqrt{{\text{k}}_{{\text{A}}_{1}{\text{B}}_{1}}^{2}+1},j\;\in \;{\text{N}}^{*}$$

According to the coordinates of the endpoints $A_{1}$ and $B_{1}$ , the straight-line equation of $A_{1}B_{1}$ is obtained, and then the straight-line equations of all other working paths are obtained according to D .

#### 3.1.2 Calculating endpoint coordinates

According to the straight-line equation of $A_{j}B_{j}$ obtained from Equation 14, determine the coordinates of the endpoints $A_{j}$ and $B_{j}$ . Make perpendicular lines $A_{1}A_{m}$ and $B_{1}B_{m}$ from point $A_{1}$ and point $B_{1}$ to line segment $A_{m}B_{m}$ (1<m≤j) , and the foot points are $A_{m}$ and $B_{m}$ . Let the slopes of line segments $A_{1}A_{m}$ and $B_{1}B_{m}$ be $k_{A_{1}A_{m}}$ and $k_{B_{1}B_{m}}$ , and the intercepts be $b_{A_{1}A_{m}}$ and $b_{B_{1}B_{m}}$ , respectively. Then the straight-line equations of $A_{1}A_{m}$ and $B_{1}B_{m}$ are expressed as,


(15) $$\{\begin{array}{c} {\text{y}}_{{\text{A}}_{1}{\text{A}}_{\text{m}}}={\text{k}}_{{\text{A}}_{1}{\text{A}}_{\text{m}}}{\text{x}}_{{\text{A}}_{1}{\text{A}}_{\text{m}}}+{\text{b}}_{{\text{A}}_{1}{\text{A}}_{\text{m}}}\\ {\text{y}}_{{\text{B}}_{1}{\text{B}}_{\text{m}}}={\text{k}}_{{\text{B}}_{1}{\text{B}}_{\text{m}}}{\text{x}}_{{\text{B}}_{1}{\text{B}}_{\text{m}}}+b_{{\text{B}}_{1}{\text{B}}_{\text{m}}} \end{array},{\text{k}}_{{\text{A}}_{1}{\text{A}}_{\text{m}}}={\text{k}}_{{\text{B}}_{1}{\text{B}}_{\text{m}}}=\frac{-1}{{\text{k}}_{{\text{A}}_{1}{\text{B}}_{1}}}$$

Substitute the coordinates $\left(x_{A_{1}},y_{A_{1}}\right)$ of point $A_{1}$ into the straight-line equation of $A_{1}A_{m}$ to obtain the intercept $b_{A_{1}A_{m}}$ of $A_{1}A_{m}$ , as shown in Equation 16.


(16) $${\text{b}}_{{\text{A}}_{1}{\text{A}}_{\text{m}}}={\text{y}}_{{\text{A}}_{1}}+\frac{{\text{x}}_{{\text{A}}_{1}}}{{\text{k}}_{{\text{A}}_{1}{\text{B}}_{1}}}$$

The straight-line equation of $A_{1}A_{m}$ can be obtained according to the intercept $b_{A_{1}A_{m}}$ and slope $k_{A_{1}A_{m}}$ . Then combine the linear equations of $A_{1}A_{m}$ and $A_{m}B_{m}$ to obtain the coordinates of the foot point $A_{m}$ as $\left(x_{A_{m}},y_{A_{m}}\right)$ , as shown in Equations 17, 18.


(17) $${\text{x}}_{{\text{A}}_{\text{m}}}=\left({\text{b}}_{{\text{A}}_{1}{\text{A}}_{\text{m}}}-{\text{b}}_{{\text{A}}_{\text{m}}{\text{B}}_{\text{m}}}\right)/\left({\text{k}}_{{\text{A}}_{\text{m}}{\text{B}}_{\text{m}}}-{\text{k}}_{{\text{A}}_{1}{\text{A}}_{\text{m}}}\right)$$

(18) $$y_{A_{m}}=k_{A_{m}B_{m}}x_{A_{m}}+b_{A_{m}B_{m}}$$

The coordinate $\left(x_{B_{m}},y_{B_{m}}\right)$ of the foot point $B_{m}$ is represented as,


(19) $${\text{x}}_{{\text{B}}_{\text{m}}}=\left({\text{b}}_{{\text{B}}_{1}{\text{B}}_{\text{m}}}-{\text{b}}_{{\text{A}}_{\text{m}}{\text{B}}_{\text{m}}}\right)/\left({\text{k}}_{{\text{A}}_{\text{m}}{\text{B}}_{\text{m}}}-{\text{k}}_{{\text{B}}_{1}{\text{B}}_{\text{m}}}\right)$$

(20) $$y_{B_{m}}=k_{A_{m}B_{m}}x_{B_{m}}+b_{A_{m}B_{m}}$$

Finally, according to Equations 14, 17-20 and the coordinates of the endpoints $A_{1}$ and $B_{1}$ , the coordinates $\left(x_{A_{m}},\;y_{A_{m}}\right)$ and $\left(x_{B_{m}},\;y_{B_{m}}\right)$ of all other endpoints can be obtained.

#### 3.1.3 Fusion of endpoints and lines

The straight-line equation of $A_{j}B_{j}$ and the coordinates of endpoints $A_{j}$ and $B_{j}$ are obtained from Sections 3.1.1 and 3.1.2, where the straight-line equation of $A_{j}B_{j}$ includes information such as slope $k_{A_{j}B_{j}}$ , intercept $b_{A_{j}B_{j}}$ , and serial number j . Use plm to represent endpoints $A_{j}$ , $B_{j}$ , and working path $A_{j}B_{j}$ , as shown in Equation 21.


(21) $$\text{plm}=\{{\text{A}}_{\text{j}},{\text{B}}_{\text{j}},{\text{A}}_{\text{j}}{\text{B}}_{\text{j}}|j\in {\text{N}}^{*}\}$$

The plm provides detailed field information for the robot, supporting the robot to obtain its own direction, serial number of working paths, and path.

### 3.2 Robots global path generation method

**Figure 3:**
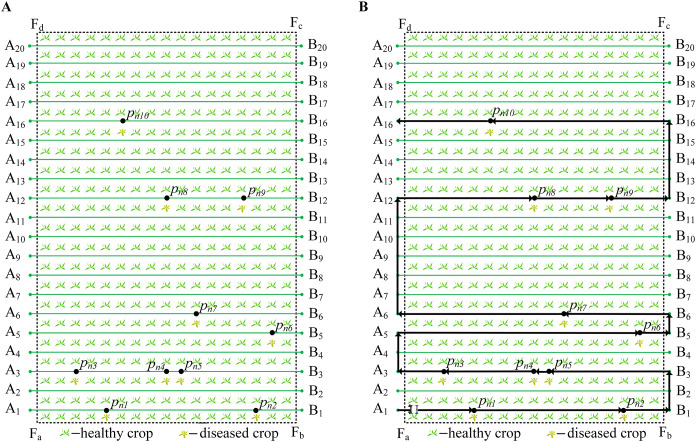
Target point and global path. **(A)** Target point. **(B)** Global path.

When a robot detects a work conflict, it needs to re-plan the path for the low-priority robot. In order to quickly plan new paths, we first use plm and target points to generate the local path of each working path. Then, all local paths are merged into the global path of the robot, as shown in **Algorithm 2**. When the robot needs to adjust its path, it only needs to change the order and direction of the local path. **Figure 3A** shows all target points of the robot. **Figure 3B** shows global path and all local paths of the robot.

Algorithm 2. Robots global path generation method 
Input: the target point  $p_{n}$
 of the robot  $R_{n}$
,  plm


Output: global path  $r^{n}$


1: **for**  i
 = 1, 2, …,  $N^{*}$

**do**

2:   **for** *j* =1, 2,…,  $N^{*}$

**do**

3:     $d_{p_{ni}A_{j}B_{j}}$


←
 the distance from point  $p_{ni}$
 to working path 

 $A_{j}B_{j}$


4:   **end for**

5:  //Get the  j
 and target point when the distance is 
the minimum value
6:   $j_{d_{min}}$
,  $p_{nd_{min}}$


←
min{ $d_{p_{ni}A_{j}B_{j}}$
}
7:   $r_{j_{d_{min}}}^{n}$


←

 $\left\{A_{j_{d_{min}}},p_{nd_{min}},B_{j_{d_{min}}}\right\}\left(0\to 1\right)$


8:   //Add local path to global path
9:   $r^{n}$


$\leftarrow r_{j_{d_{min}}}^{n}$


10: **end for**

11: **for**  $r_{j}^{n}$
 **in**  $r^{n}$
 **do**

12:  //Merge local paths with the same  j


13:   $r_{j}^{n}\leftarrow \left\{A_{j},p_{n1},\;p_{n2},\ldots ,p_{nv},B_{j}\right\}\left(0\to 1\right)$


14: **end for**

15: Update global path  $r^{n}$
 according to the modified 
$r_{j}^{n}$


16: **for** 
$r_{j}^{n}$
 **in**  $r^{n}$
 **do**

17:    $d_{p_{nv}A_{j}}$
 
←
 the distance from point  $p_{nv}$
 to working path 
$A_{j}$


18:  //Sort the target points  $\left\{p_{n1},\;p_{n2},\ldots ,p_{nv}\right\}$
 according 
to the size of  $d_{p_{nv}A_{j}}$


19:   $r_{j}^{n}\leftarrow \left\{A_{j},p_{n1},\;p_{n2},\ldots ,p_{nv},B_{j}\right\}\left(0\to 1\right)$
, 
$d_{p_{n1}A_{j}}<d_{p_{n2}A_{j}}<\cdots <d_{p_{nv}A_{j}}$
,  0<v≤i


20: **end for**

21: Update global path 
$r^{n}$
 according to the modified 
$r_{j}^{n}$


22://According to the requirements of direction 
(0→1)(1→0)(0→1)⋯
, adjust the 
$r^{n}$


23: 
$r^{n}\;\leftarrow \left\{r_{1}^{n}\left(0\to 1\right),r_{2}^{n}\left(1\to 0\right),r_{3}^{n}\left(0\to 1\right),\ldots ,r_{j}^{n}\left(1\to 0\right)\right\}$


24: **return** *r^n^
*

#### 3.2.1 The local path of the robot

According to the plm and target point $p_{n}=\left\{p_{n1},\;p_{n2},\ldots ,p_{ni}\right\}$ of the robot $R_{n}$ , plan the local path $r_{j}^{n}$ of the robot. Let the coordinates of $p_{ni}$ be $\left(x_{p_{ni}},y_{p_{ni}}\right)$ , and calculate the distance $d_{p_{ni}A_{j}B_{j}}$ from point $p_{ni}$ to straight line $A_{j}B_{j}$ according to Equation 22.


(22) $$d_{p_{ni}A_{j}B_{j}}=\frac{\left|k_{A_{j}B_{j}}-y_{p_{ni}}+b_{A_{j}B_{j}}\right|}{\sqrt{1+k_{A_{j}B_{j}}^{2}}}\;,A_{j}B_{j}\;\in \;plm,i\;\in \;N^{*}$$

Let $j_{d_{min}}$ represent the serial number of the working path when $d_{p_{ni}A_{j}B_{j}}$ takes the minimum value, as shown in Equation 23. When $d_{p_{ni}A_{j}B_{j}}$ takes the minimum value, the target point $p_{ni}$ is in the working path

$A_{j_{d_{min}}}B_{j_{d_{min}}}$ .


(23) $$j_{d_{min}}=\{j|min\{d_{p_{ni}A_{j}B_{j}}|i\;\in \;N^{*},A_{j}B_{j}\;\in \;plm\}\}$$

The endpoints $A_{j_{d_{min}}}$ and $B_{j_{d_{min}}}$ of the working path $A_{j_{d_{min}}}B_{j_{d_{min}}}$ and point $p_{ni}$ constitute the local path $r_{j_{d_{min}}}^{n}$ , as shown in Equation 24. Put $p_{ni}$ between points $A_{j_{d_{min}}}$ and $B_{j_{d_{min}}}$ because the robot starts from the endpoint $A_{j_{d_{min}}}$ or $B_{j_{d_{min}}}$ and then passes through the target point

$p_{ni}$ .


(24) $$\begin{array}{c} r_{j_{d_{min}}}^{n}=\left\{A_{j_{d_{min}}},p_{ni},B_{j_{d_{min}}}\right\}\\ r_{j_{d_{min}}}^{n}=\left\{B_{j_{d_{min}}},p_{ni},A_{j_{d_{min}}}\right\} \end{array}$$

Calculate the distance $d_{p_{nv}A_{j}}(0<v\leq i)$ from point $p_{ni}$ to $A_{j}$ according to Equation 25 when two or more target points are in the same working path.


(25) $$d_{p_{nv}A_{j}}=\sqrt{{\left(x_{p_{nv}}-x_{A_{j}}\right)}^{2}+{\left(y_{p_{nv}}-y_{A_{j}}\right)}^{2}},\;\left(0<v\leq i\right),A_{j}B_{j}\;\in \;plm$$

Sort the target points $\left\{p_{n1},\;p_{n2},\ldots ,p_{nv}\right\}$ according to the size of $d_{p_{nv}A_{j}}$ , and then update the local path $r_{j}^{n}$ , as shown in Equation 26.


(26) $$\begin{array}{l} r_{j}^{n}=\left\{A_{j},p_{n1},\;p_{n2},\ldots ,p_{nv},B_{j}\right\},\;d_{p_{n1}A_{j}}<d_{p_{n2}A_{j}}<\ldots <d_{p_{nv}A_{j}}\\ r_{j}^{n}=\left\{B_{j},p_{n1},\;p_{n2},\ldots ,p_{nv},A_{j}\right\},d_{p_{n1}A_{j}}>d_{p_{n2}A_{j}}>\ldots >d_{p_{nv}A_{j}} \end{array}$$

In **Figure 3A**, $p_{n1}$ and $p_{n2}$ are in the working path $A_{1}B_{1}$ . Since $d_{p_{n1}A_{1}}<d_{p_{n2}A_{1}}$ , the path $r_{1}^{n}=\left\{A_{1},p_{n1},p_{n2},B_{1}\right\}$ is obtained.

According to the target point $p_{n}$ , a series of local paths $r_{1}^{n}$ , $r_{2}^{n}$ , $r_{3}^{n}$ ,…, $r_{j}^{n}$ are obtained. For example, **Figure 3A** contains target points $\;\{p_{n1},p_{n2},\ldots ,p_{n10}\}$ , and six groups of local paths are obtained, as shown in Equation 27.


(27) $$\begin{array}{l} r_{1}^{n}=\left\{A_{1},p_{n1},p_{n2},B_{1}\right\}\left(0\to 1\right)\\ r_{3}^{n}=\left\{A_{3},p_{n3},p_{n4},p_{n5},B_{3}\right\}\left(0\to 1\right)\\ r_{5}^{n}=\left\{A_{5},p_{n6},B_{5}\right\}\left(0\to 1\right)\\ r_{6}^{n}=\left\{A_{6},p_{n7},B_{6}\right\}\left(0\to 1\right)\\ r_{12}^{n}=\left\{A_{12},p_{n8},p_{n9},B_{12}\right\}\;\left(0\to 1\right)\\ r_{16}^{n}=\left\{A_{16},p_{n10},B_{16}\right\}\left(0\to 1\right) \end{array}$$

#### 3.2.2 Robots global path generation method

According to the local path $r_{j}^{n}$ of the robot, it is known that the serial number of the working path is j . Sort the local paths $r_{j}^{n}$ according to j and then get the path $r^{n}=\left\{r_{1}^{n},r_{2}^{n},r_{3}^{n},\ldots ,r_{j}^{n}\right\}$ . For example, the path sorting in **Figure 3A** results in $r^{n}=\left\{r_{1}^{n},r_{3}^{n},r_{5}^{n},r_{6}^{n},r_{12}^{n},r_{16}^{n}\right\}$ .

However, the sorted $r^{n}$ cannot be directly used as a robot’s global path because the connection between local paths is not continuous. For example, $r_{1}^{n}$ to $r_{3}^{n}$ in Equation 27 is from point $B_{1}$ to $A_{3}$ and finally to $B_{3}$ , which does not conform to the ‘U’ shape path of the robot. The robot cannot go directly from $B_{1}$ to $A_{3}$ but should go from $B_{1}$ to $B_{3}$ , then to $A_{3}$ . Therefore, it is necessary to adjust $r_{3}^{n}$ to $\left\{B_{3},.,A_{3}\right\}$ , and at the same time, reorder the target points inside $r_{3}^{n}$ according to Equation 25 to obtain the updated $r_{3}^{n}=\left\{B_{3},p_{n5},p_{n4},p_{n3},A_{3}\right\}\left(1\to 0\right)$ . Similarly, update $r_{5}^{n}$ , $r_{6}^{n}$ , $r_{12}^{n}$ , $r_{16}^{n}$ according to this rule, and finally update the six groups of local paths in Equation 27 to get Equation 28.


(28) $$\begin{array}{l} r_{1}^{n}=\left\{A_{1},p_{n1},p_{n2},B_{1}\right\}\left(0\to 1\right)\\ r_{3}^{n}=\left\{B_{3},p_{n5},p_{n4},p_{n3},A_{3}\right\}\left(1\to 0\right)\\ r_{5}^{n}=\left\{A_{5},p_{n6},B_{5}\right\}\left(0\to 1\right)\\ r_{6}^{n}=\left\{B_{6},p_{n7},A_{6}\right\}\left(1\to 0\right)\\ r_{12}^{n}=\left\{A_{12},p_{n8},p_{n9},B_{12}\right\}\left(0\to 1\right)\\ r_{16}^{n}=\left\{B_{16},p_{n10},A_{16}\right\}\left(1\to 0\right) \end{array}$$

Therefore, to ensure that the local paths are continuous, the direction between the local paths must satisfy (0→1) (1→0) (0→1)⋯  or (1→0) (0→1) (1→0)⋯ . $r^{n}$ satisfies Equation 29 or 30.


(29) $$r^{n}=\left\{r_{1}^{n}\left(0\to 1\right),r_{2}^{n}\left(1\to 0\right),r_{3}^{n}\left(0\to 1\right),\ldots ,r_{j}^{n}\left(1\to 0\right)\right\}$$

(30) $$r^{n}=\left\{r_{1}^{n}\left(1\to 0\right),r_{2}^{n}\left(0\to 1\right),r_{3}^{n}\left(1\to 0\right),\ldots ,r_{j}^{n}\left(0\to 1\right)\right\}$$

Combining the six groups of local paths in Equation 28, the global path $r^{n}$ is obtained, as shown in Equation 31. Finally, according to the target point $p_{n}=\left\{p_{n1},p_{n2},\ldots ,p_{n10}\right\}$ of the robot $R_{n}$ in **Figure 3A**, plan the black path $r^{n}$ in **Figure 3B**.


(31) $$r^{n}=\left\{r_{1}^{n}\left(0\to 1\right),r_{3}^{n}\left(1\to 0\right),r_{5}^{n}\left(0\to 1\right),r_{6}^{n}\left(1\to 0\right),r_{12}^{n}\left(0\to 1\right),r_{16}^{n}\left(1\to 0\right)\right\}$$

### 3.3 Itinerary table with all global information

Based on the plm and its own position, the robot can calculate the direction of the robot, the number of the current working path, and the type of path. Combined with the global path and priority, the robot can learn all global information. When robot $R_{n}$ learns its own information, it needs to send its global information to other robots so that other robots can understand the status of $R_{n}$ , thereby judging the relationship between robots and predicting the possibility of collision. We designed an itinerary table (as shown in **Table 1**) that contains the robot’s serial number, priority, path type, moving direction, real-time position, target position, current serial number of the working path and last serial number of the working path to facilitate the robot’s sending and receive. When robot $R_{n}$ is started, it generates an itinerary table $H_{R_{n}}$ .` H represents the itinerary table of a group of working robots, and $H_{R_{n}}$ represents the itinerary table of robot $R_{n}$ , as shown in Equation 32.

**Table 1:** The itinerary table of robot $R_{n}$ .

Itinerary table	
serial number of the robot	0, 1, 2, 3, …, n
priority	0, 1, 2, 3, …
path type	working or transition path
moving direction	0→1 , 1→0 or up , down
real-time position	$P_{R_{n}}$
target position	$P_{n}$
current serial number of the working path	$j_{c}$
last serial number of the working path	$j_{l}$


(32) $$H=\{H_{R_{n}}|n\;\in \;N^{*}\}$$

$H_{O}$ represents other itinerary tables except for $H_{R_{n}}$ , as shown in Equation 33.


(33) $$H_{O}=H-H_{R_{n}}$$

Robot $R_{n}$ constantly updates the data in the itinerary table while working and broadcasts its $H_{R_{n}}$ to other robots $R_{O}$ . At the same time, robot $R_{n}$ receives $H_{O}$ and makes decisions.

### 3.4 Multi-robot collision avoidance method

Based on the point-line map, global path and itinerary table, the robot can understand the global information of the field and the status of other robots. With this information, the robot can make reasonable decisions and avoid collisions with other robots. The multi-robot collision avoidance algorithm is shown in **Algorithm 3**. We calculate the direction of the robot $R_{n}$ in different path type through Equations 5, 6. Then obtain the moving direction of other robots through the received itinerary table $H_{O}$ . Finally, we can compare whether the robots have the same direction.

Algorithm 3. Multi-robot collision avoidance method 
Input:  $r^{n}$
,  $H_{R_{n}}$


1: **while** true **do**

2:  Calculate the directions of robots according to Equations 5, 6.
3:  **if** the robots move in the same direction **then**

4:    $d_{R_{n}R_{o}}$
  ←
 the distance from robot  $R_{n}$
 to  $R_{O}$


5:   **if** 
$d_{R_{n}R_{o}}<1$
 **then**

6:    The robot at the rear stops moving
7:   **else**

8:    The robot keeps moving
9:   **end if**

10: **else**

11:  //According to the local path  $r_{j}^{n}$
, query the path type
12:  If the robot path points from  $A_{j}$
 to  $B_{j}$
 or  $B_{j}$
 to  $A_{j}$
, the path type is working path.
13:  If the robot path points from  $A_{s}$
 to  $A_{u}$
 or  $B_{s}$
 to  $B_{u}$
, the path type is transition path.
14:  **if** two or more robots have the same path type **then**

15:    **if** path type is working path **then**

16:      $j_{c}$
  ← 
 get the serial number of the working path based on the local path  $r_{j}^{n}$


17:     **if**  $j_{c}$
 is the same **then**

18:      //Adjust low-priority robot paths
19:      $r^{n}=\left\{r_{j}^{n}|j\in N^{*}\right\}\to r^{n}=\left\{r_{j}^{n},r_{s}^{n}|j\in \left(N^{*}-s\right)\right\}$


20:      Then, the robot continues to move along the new path
21:     **else**

22:      The robot continues to move along the global path
23:     **end if**

24:    **else if** path type is transition path **then**

25:      $d_{R_{n}R_{o}}$
  ←
 the distance from robot  $R_{n}$
 to  $R_{O}$


26:     **if** 
 $d_{R_{n}R_{o}}<1$
 **then**

27:     //Add obstacle avoidance paths to the  $r^{n}$
 of low-priority robot.
28:       $r^{n}=\left\{r_{j}^{n}|j\in N^{*}\right\}\to r^{n}=\left\{\ldots ,r_{s}^{n},r_{t}^{n},r_{u}^{n},\ldots |t\rangle j,s\neq u,0<s\leq j,0<u\leq j\right\}$ 
29:      Then, the robot continues to move along the new path
30:     **else**

31:      The robot continues to move along the global path
32:     **end if**

33:    **end if**

34:   **else**

35:    The robot continues to move along the global path
36:   **end if**

37:  **end if**

38:  **if** 
 $P_{R_{n}}$
 is the last point in the target point  $P_{n}$
 **then**

39:   **break**

40:  **end if**

41: **end while**

If the direction is the same, query the real-time position of the robot with the same direction in the itinerary table $H_{O}$ , and calculate the distance $d_{R_{n}R_{o}}$ between $R_{n}$ and $R_{O}$ . When $d_{R_{n}R_{o}}\geq 1$ , $R_{n}$ and $R_{O}$ maintain the current moving state. When $d_{R_{n}R_{o}}<1$ , the robot at the rear stops moving, and when $d_{R_{n}R_{o}}\geq 1$ , the robot at the rear resumes moving.

But if the directions are different, the robot must make different decisions depending on the path type. We determine the path type of the robot based on its local path. If the robot path is from $A_{j}$ to $B_{j}$ or $B_{j}$ to $A_{j}$ , the path type of the robot is a working path. If the robot’s path is from $A_{s}$ to $A_{u}$ or $B_{s}$ to $B_{u}$ , the path type of the robot is a transition path.

#### 3.4.1 On the working path

If the path types of multiple robots are working path, we query the current serial number of the working path of the robot through the local path $r_{j}^{n}$ .If the serial number $j_{c}$ is different, $R_{n}$ and $R_{O}$ are not in the same working path, and there is no possibility of conflict. Robots continue to move according to the global path and do not need to do anything.

If the serial number $j_{c}$ is the same, $R_{n}$ and $R_{O}$ are in the same working path, and conflicts or collisions will occur. There are two conflict cases: The first case is that one robot is moving in the working path, and another robot is moving from the transition path to the working path; The second is that all robots are entering the working path from the transition path.

For the first case, for example, when $R_{O}$ is already moving in the working path $A_{s}B_{s}$ , $R_{n}$ is moving from $A_{s}A_{u}$ to $A_{s}B_{s}$ . Since they are going in different directions, they must collide. We do not consider the priorities of the robots at this time, directly let the robot $R_{n}$ give up the current working path, and then re-plan the global path. In order not to hinder the robot’s work and quickly generate the remaining path of the robot, we move the current conflicting local path $r_{s}^{n}$ to the end of the global path, as shown in Equation 34. Then, using the content of Section 3.2.2, according to the direction continuity between local paths, modify the target point order of the local path, and then update $r^{n}$ . The robot $R_{n}$ continues to move according to the updated $r^{n}$ .


(34) $$r^{n}=\left\{r_{j}^{n}|j\;\in \;N^{*}\right\}\to r^{n}=\left\{r_{j}^{n},r_{s}^{n}|j\;\in \;\left(N^{*}-s\right)\right\}$$

For the second case, for example, both $R_{O}$ and $R_{n}$ are moving on the transition path and have not yet reached the working path. At this time, consider the priority of $R_{O}$ and $R_{n}$ , assuming that the priority of $R_{n}$ is lower than $R_{O}$ . The robot $R_{n}$ with low priority abandons the current working path $A_{s}B_{s}$ and re-plans the path. The remaining path re-planning rules are the same as in the first case.

#### 3.4.2 On the transition path

If multiple robots are on a transition path, we first query the real-time positions of the robots on the same path type. Then, calculate the distance $d_{R_{n}R_{o}}$ between the robots. When $d_{R_{n}R_{o}}>1$ , robots maintain the current moving state. When $d_{R_{n}R_{o}}\leq 1$ , the robot with low priority avoids the one with high priority. Since the width of the transition path is generally relatively wide, multiple robots can be accommodated simultaneously in the lateral direction. We can add some extra paths to avoid robot collisions.

**Figure 4:**
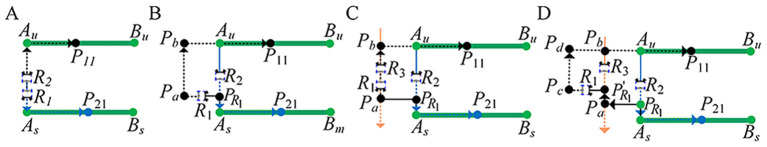
Conflict detection for robots on transition paths. **(A)** $R_{1}$ and $R_{2}$ meet at the transition path. **(B)** $R_{1}$ plans a new path. **(C)** $R_{1}$ meets $R_{3}$ on the new path. **(D)** $R_{1}$ plans a new path again.


In **Figure 4A**, robot $R_{1}$ goes from point $A_{s}$ to $P_{11}$ , robot $R_{2}$ goes from point $A_{u}$ to $P_{21}$ . It is obvious that $R_{1}$ and $R_{2}$ have different directions. R1 and R2 are close together and are about to collide. Assuming that robot $R_{1}$ has a lower priority than robot $R_{2}$ . The low-priority robot translates the current local path a certain distance to the left or right to avoid collision. For example, in **Figure 4B**, robot $R_{1}$ translates the local path $P_{R_{n}}A_{u}$ to $P_{a}P_{b}$ to avoid collision with $R_{2}$ . After translating the local path, the path $P_{a}P_{b}$ is updated to { $P_{R_{n}},P_{a},P_{b},A_{u}$ } according to the continuity of the path. We set the new path to $r_{t}^{1}$ , as shown in Equation 35.

(35) $$r_{t}^{1}=\left\{P_{R_{n}},P_{a},P_{b},A_{u}\right\},t>j$$

The conflict occurs on the transition path $A_{s}A_{u}$ , and $A_{s}A_{u}$ is located between the local paths $r_{\text{s}}^{1}$ and $r_{u}^{1}$ , so insert $r_{\text{t}}^{1}$ between $r_{\text{s}}^{1}$ and $r_{u}^{1}$ , as shown in Equation 36. The moving direction of the robot on the transition path $A_{s}A_{u}$ is up or down , and it is 0→1 or 1→0 on the working paths $A_{s}B_{s}$ and $A_{u}B_{u}$ , so insert $r_{\text{t}}^{1}$ between $r_{s}^{1}$ and $r_{u}^{1}$ , and the direction between other local paths in $r^{1}$ still satisfies (0→1) (1→0) (0→1) ⋯ or (1→0) (0→1) (1→0 ) ⋯ . Therefore, we only need to simply insert the local obstacle avoidance path $r_{\text{t}}^{1}$ between $r_{\text{s}}^{1}$ and $r_{u}^{1}$ without doing other operations.


(36) $$r^{1}=\left\{\ldots ,r_{s}^{1},r_{t}^{1},r_{u}^{1},\ldots \right\},s\neq u,0<s\leq j,0<u\leq j$$

When a new robot is detected, the low-priority robot continues to translate the local path left or right. In **Figure 4C**, robot $R_{3}$ is detected on path $P_{a}P_{b}$ after $R_{1}$ passes through point $P_{a}$ , and robot $R_{1}$ plans local avoidance path $r_{t_{1}}^{1}$ , as shown in Equation 37.


(37) $$r_{t_{1}}^{1}=\left\{p_{R_{n}}^{\prime },P_{c},P_{d},P_{b},A_{u}\right\}$$

Update $r_{t}^{1}\;$ according to the local avoidance path $r_{t_{1}}^{1}$ , as shown in Equation 38. Then insert the updated $r_{t}^{1}$ into the global path $r^{1}$ .


(38) $$r_{t}^{1}=\left\{P_{R_{n}},P_{a},p_{R_{n}}^{\prime },P_{c},P_{d},P_{b},A_{u}\right\}$$

The updated local avoidance path is shown in **Figure 4D**. If $R_{1}$ conflicts with other robots again, plan the local obstacle avoidance path according to this rule and then update $r_{t}^{1}$ and $r^{1}$ .

## 4 Experiment and result

### 4.1 Simulate initial conditions

**Figure 5:**
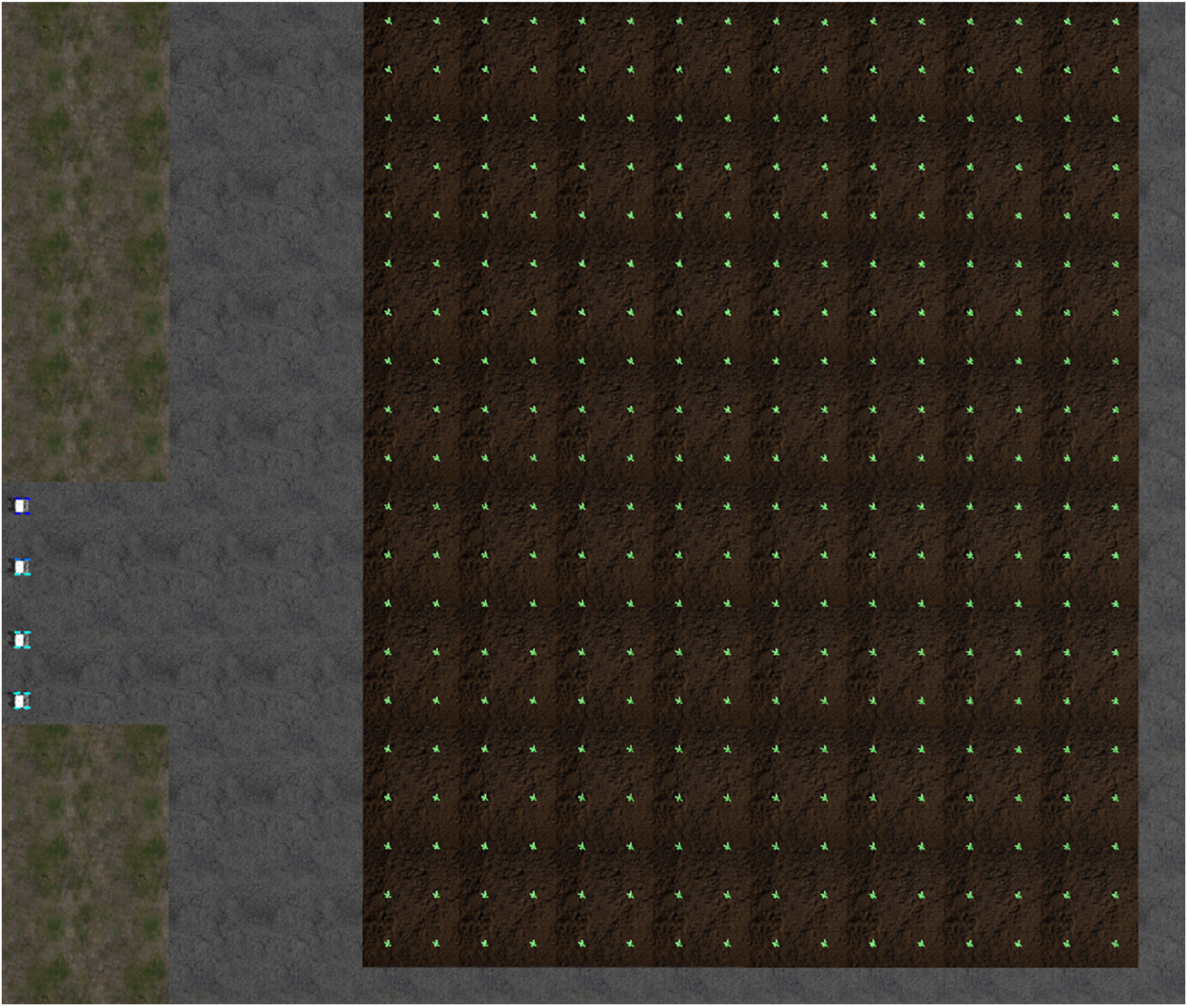
The robot’s simulation work environment.

In order to verify our proposed multi-robot collision avoidance method, we used Gazebo software to design the robot working environment. The working environment includes field, crops, roads, and four working robots, as shown in **Figure 5**. Each working robot contains a medicine storage barrel, a medicine spraying device, communication device, positioning device and controller.

**Figure 6:**
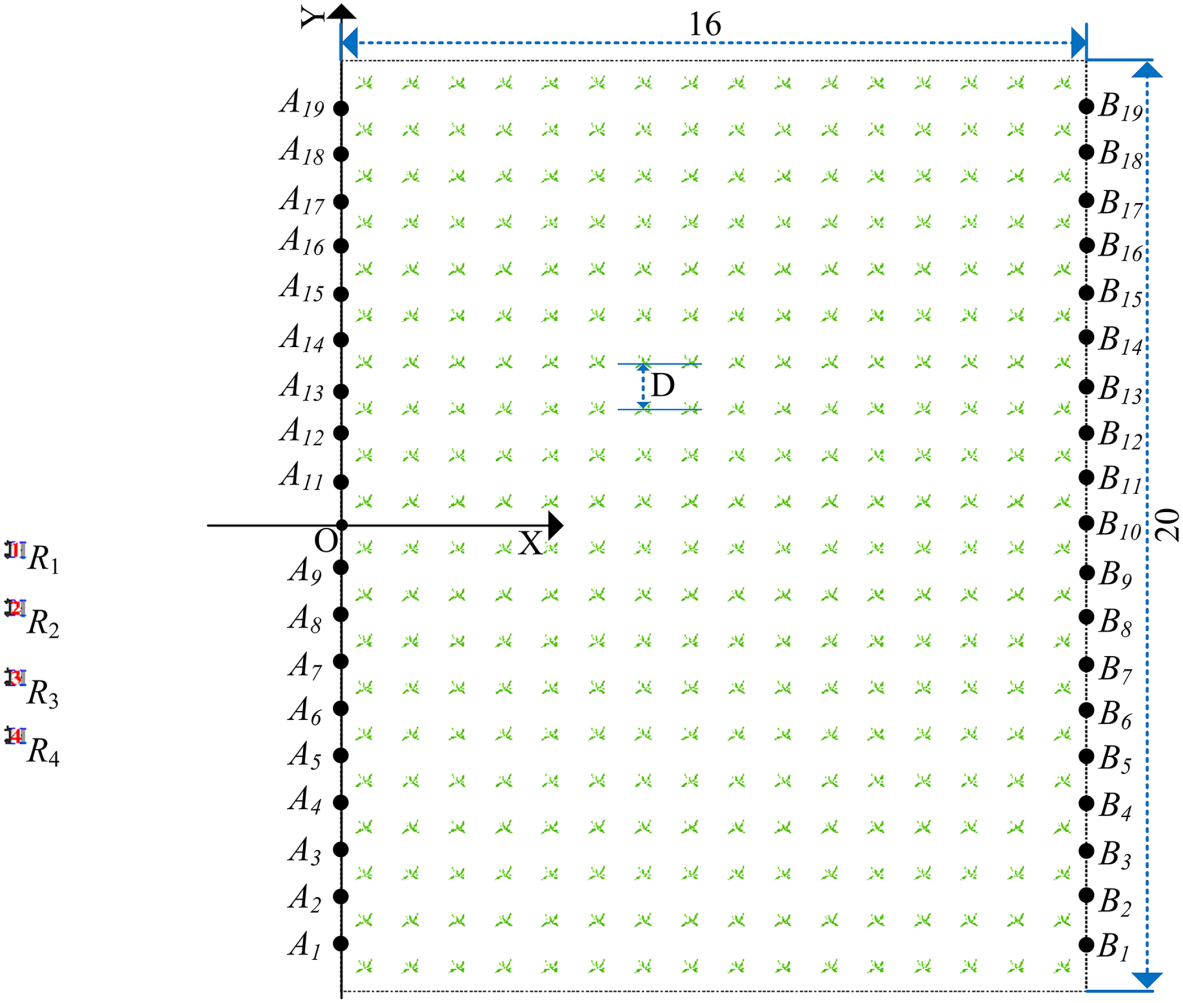
The initial diagram of the robot’s working environment.

In order to facilitate the description of the working process of the robot, a GUI display interface is designed to display the garage, field, crops, and robot, as shown in **Figure 6**. Set up a group of working robots $R=\left\{R_{1},R_{2},R_{3},R_{4}\right\}$ , with priorities of 0, 1, 2, and 3, respectively. Set the field length to 20 meters and width to 16 meters. The field has 20 rows of crops ( 1≤j<20 ), and the distance between each row of crops is 1 meter ( D=1 ). The coordinate system is established with point O as the origin, and the coordinates of the two endpoints $A_{1}$ and $B_{1}$ of the baseline $A_{1}B_{1}$ are set to (0, -9) and (16, -9), respectively.

### 4.2 Generating a point-line map

We first generate all working path $A_{j}B_{j}$ and endpoints $A_{j}$ and $B_{j}$ based on the baseline $A_{1}B_{1}$ . Calculate the slope $k_{A_{1}B_{1}}$ and intercept $b_{A_{1}B_{1}}$ of the working path $A_{1}B_{1}$ based on points $A_{1}$ and $B_{1}$ and the straight-line equation of $A_{1}B_{1}$ , as shown in Equation 39.


(39) $$y_{A_{1}B_{1}}=-9\left(0\leq x_{A_{1}B_{1}}\leq 16\right),k_{A_{1}B_{1}}=0,b_{A_{1}B_{1}}=-9$$

According to Equations 14, 39, calculate the straight-line equation of the working path $A_{j}B_{j}$ , as shown in Equation 40.


(40) $$y_{A_{j}B_{j}}=-9+\left(j-1\right)*D,k_{A_{j}B_{j}}=0,b_{A_{j}B_{j}}=-9+\left(j-1\right)*D$$

Then according to Equations 17-20, the coordinates of points $A_{j}$ and $B_{j}$ are obtained as (0,−9+(j−1)*D) , ( 16,−9+(j−1)*D) . Finally, according to Equations 39, 40, the plm is obtained as shown in Equation 41.


(41) $$\begin{array}{c} plm=\{A_{j}\left(0,-9+\left(j-1\right)*D\right),B_{j}\left(16,-9+\left(j-1\right)*D\right),\\ y_{A_{j}B_{j}}=-9+\left(j-1\right)*D|1\leq j<20,D=1\} \end{array}$$

### 4.3 Generating the global path

**Figure 7:**
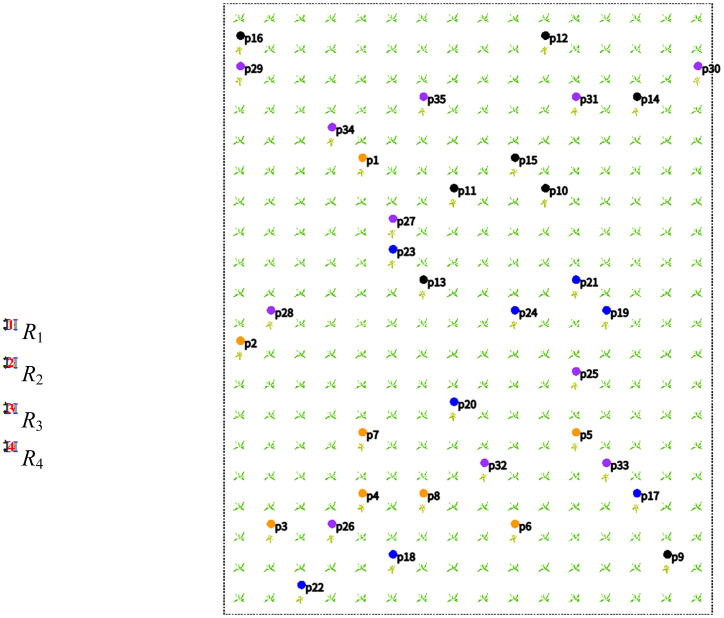
Random distribution of target points.

We randomly generated the coordinates of 35 diseased crops, and then used them as the target points of the robot (Meshram et al., 2022). The target point is denoted as $P=\left\{p_{1},p_{2},p_{3},\ldots ,p_{35}\right\}$ . Then, the coordinates P are randomly assigned to $R_{1}$ , $R_{2}$ , $R_{3}$ , and $R_{4}$ , as shown in **Figure 7**. Randomly divide the targets into four groups: $P_{1}=\left\{p_{11},p_{12},\ldots ,p_{18}\right\}$ is represented by orange, $P_{2}=\left\{p_{29},p_{210},\ldots ,p_{216}\right\}$ is represented by black, $P_{3}=\left\{p_{317},p_{318},\ldots ,p_{324}\right\}$ is represented by blue, and $P_{4}=\left\{p_{424},p_{425},\ldots ,p_{435}\right\}$ is represented by purple.

According to Section 3.2, use the target points and map to generate the robots’ global path, as shown in Equations 42-45.


(42) $$r^{1}=\left\{r_{15}^{1}\left(0\to 1\right),r_{9}^{1}\left(1\to 0\right),r_{6}^{1}\left(0\to 1\right),r_{4}^{1}\left(1\to 0\right),r_{3}^{1}\left(0\to 1\right)\right\}$$

where $r_{15}^{1}=\left\{A_{15},p_{11},B_{15}\right\}$ , $r_{9}^{1}=\left\{B_{9},p_{12},A_{9}\right\}$ , $r_{6}^{1}=\left\{A_{6},p_{17},p_{15},B_{6}\right\}$ , $r_{4}^{1}=\left\{B_{4},p_{18},p_{14},A_{4}\right\}$ , $r_{3}^{1}=\left\{A_{3},p_{13},p_{16},B_{3}\right\}$ .


(43) $${\text{r}}^{2}=\left\{{\text{r}}_{2}^{2}\left(0\to 1\right),{\text{r}}_{11}^{2}\left(1\to 0\right),{\text{r}}_{14}^{2}\left(0\to 1\right),{\text{r}}_{15}^{2}\left(1\to 0\right),{\text{r}}_{17}^{2}\left(0\to 1\right),{\text{r}}_{19}^{2}\left(1\to 0\right)\right\}$$

where $r_{2}^{2}=\left\{A_{2},p_{29},B_{2}\right\}$ , $r_{11}^{2}=\left\{B_{11},p_{213},A_{11}\right\}$ , $r_{14}^{2}=\left\{A_{14},p_{211},p_{210},B_{14}\right\}$ , $r_{15}^{2}=\left\{B_{15},p_{215},A_{15}\right\}$ , $r_{17}^{2}=\left\{A_{17},p_{214},B_{17}\right\}$ , $r_{19}^{2}=\left\{B_{19},p_{212},p_{216},A_{19}\right\}$ .


(44) $$\begin{array}{c} {\text{r}}^{3}=\{{\text{r}}_{1}^{3}\left(0\to 1\right),{\text{r}}_{2}^{3}\left(1\to 0\right),{\text{r}}_{4}^{3}\left(0\to 1\right),{\text{r}}_{7}^{3}\left(1\to 0\right),\\ {\text{r}}_{10}^{3}\left(0\to 1\right),{\text{r}}_{11}^{3}\left(1\to 0\right),{\text{r}}_{12}^{3}\left(0\to 1\right)\} \end{array}$$

where $r_{1}^{3}=\left\{A_{1},p_{322},B_{1}\right\}$ , $r_{2}^{3}=\left\{B_{2},p_{318},A_{2}\right\}$ , $r_{4}^{3}=\left\{A_{4},p_{317},B_{4}\right\}$ , $r_{7}^{3}=\left\{B_{7},p_{320},A_{7}\right\}$ , $r_{10}^{3}=\left\{A_{10},p_{324},p_{319},B_{10}\right\}$ , $r_{11}^{3}=\left\{B_{11},p_{321},A_{11}\right\}$ , $r_{12}^{3}=\left\{A_{12},p_{323},B_{12}\right\}$ .


(45) $$\begin{array}{c} {\text{r}}^{4}=\{r_{3}^{4}\left(0\to 1\right),{\text{r}}_{5}^{4}\left(1\to 0\right),{\text{r}}_{8}^{4}\left(0\to 1\right),{\text{r}}_{10}^{4}\left(1\to 0\right),\\ r_{13}^{4}\left(0\to 1\right),{\text{r}}_{16}^{4}\left(1\to 0\right),r_{17}^{4}\left(0\to 1\right),{\text{r}}_{18}^{4}\left(1\to 0\right)\} \end{array}$$

where $r_{3}^{4}=\left\{A_{3},p_{426},B_{3}\right\}$ , $r_{5}^{4}=\left\{B_{5},p_{433},p_{432},A_{5}\right\}$ , $r_{8}^{4}=\left\{A_{8},p_{425},B_{8}\right\}$ , $r_{10}^{4}=\left\{B_{10},p_{428},A_{10}\right\}$ , $r_{13}^{4}=\left\{A_{13},p_{427},B_{13}\right\}$ , $r_{16}^{4}=\left\{B_{16},p_{434},A_{16}\right\}$ , $r_{17}^{4}=\left\{A_{17},p_{435},p_{431},B_{17}\right\}$ , $r_{18}^{4}=\left\{B_{18},p_{429},A_{18}\right\}$ .

**Figure 8:**
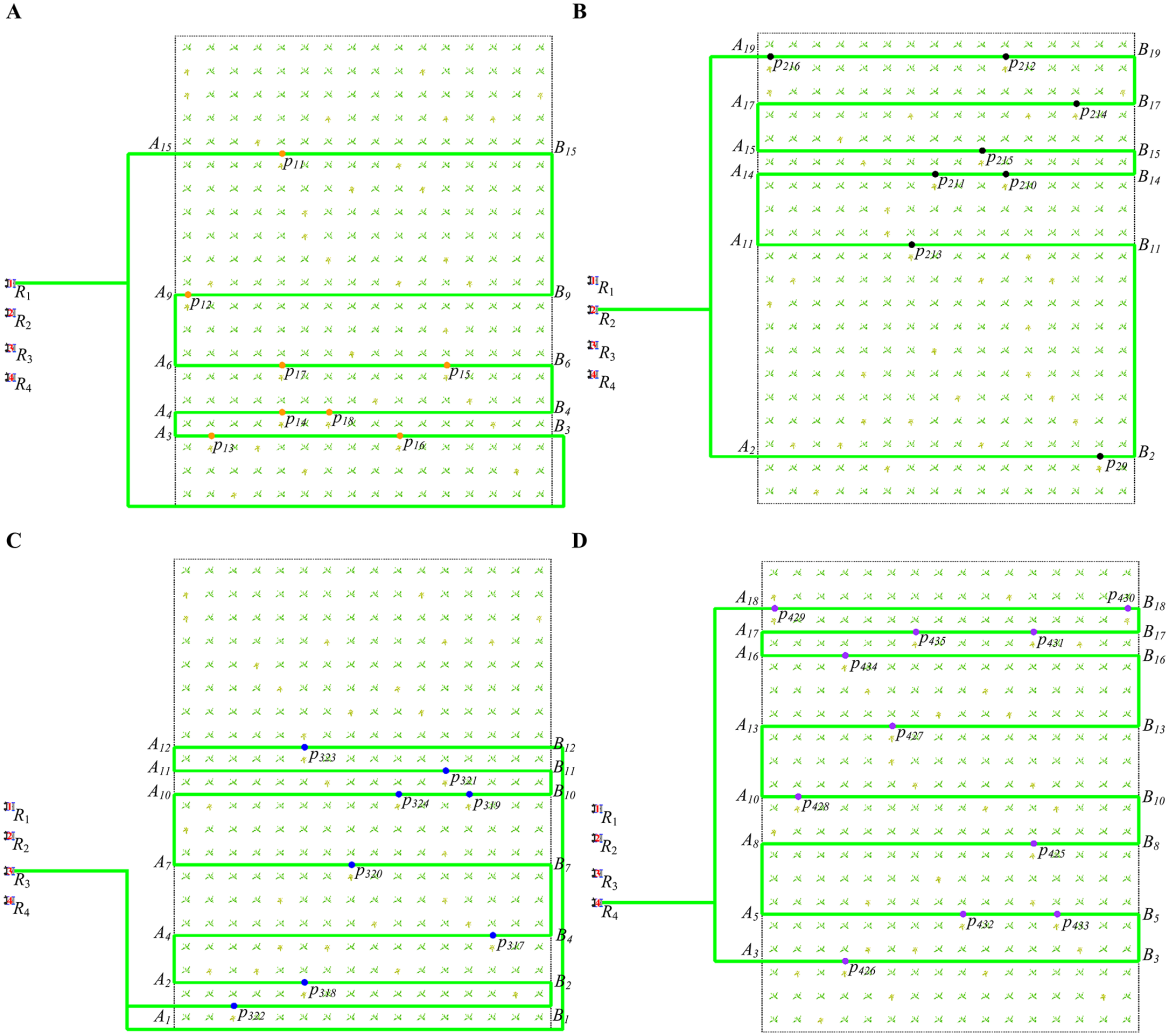
The global path of four robots. **(A)** The global path of $R_{1}$ . **(B)** The global path of $R_{2}$ . **(C)** The global path of $R_{3}$ . **(D)** The global path of $R_{4}$ .

**Figures 8A–D** show the global paths of $R_{1}$ , $R_{2}$ , $R_{3}$ , and $R_{4}$ .

### 4.4 Simulation experiment of multi-robot collision avoidance method

After planning the global path, the robots start spraying operations and simultaneously create an itinerary table H , as shown in **Table 1**. During the operation, the robot determines the position, path, and direction of moving of other robots by querying their itinerary tables. Then, the robots detect four types of collision and conflict according to section 2.2. Next, the multi-robot collision avoidance method is simulated considering four types of collisions and conflicts.

#### 4.4.1 Working path and different movement directions

When the serial number $j_{c}$ of the working path is the same, and the moving direction is different, there are two conflict cases: The first case is that one robot is moving in the working path, and another robot is moving from the transition path to the working path; The second is that all robots are entering the working path from the transition path.

**Figure 9:**
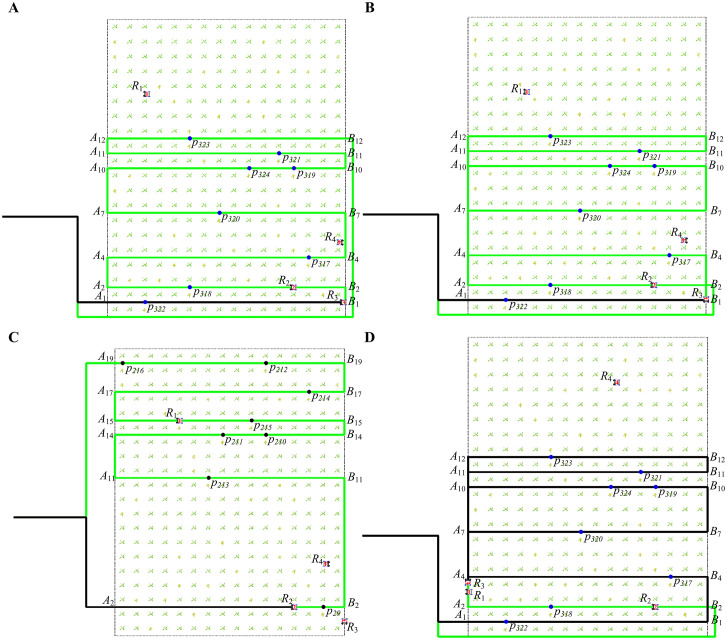
The paths of robots moving in different directions on the same working path at different times (the first case). **(A)** The path of robot $R_{3}$ at 121 seconds. **(B)** The path of robot $R_{3}$ at 125 seconds. **(C)** The path of robot $R_{2}$ at 125 seconds. **(D)** The path of robot $R_{3}$ at 522 seconds.

**Figure 9** shows the first case (The green path represents the path that has not been passed, while the black path represents the path that has already been passed). Robot $R_{2}$ is moving in the working path $A_{2}B_{2}$ , and robot $R_{3}$ is moving from the transition path $B_{1}B_{2}$ to the working path $A_{2}B_{2}$ . **Figure 9A** shows that at 121 seconds, robot $R_{3}$ is about to leave its current working path $A_{1}B_{1}$ and move to the next working path $A_{2}B_{2}$ for work. **Figure 9B** shows that at 125s, robot $R_{3}$ moves to the transition path $B_{1}B_{2}$ and detects a robot $R_{2}$ (At 25 seconds, $R_{2}$ works on working path $A_{2}B_{2}$ , as shown in **Figure 9C**) with a different direction of moving in the working path $A_{2}B_{2}$ according to the itinerary table.

In order to avoid collision, robot $R_{2}$ will abandon the current local path $r_{2}^{3}$ and then re-plan the remaining path. We follow the method proposed in Section 3 and first move the current local path $r_{2}^{3}$ to the end of the global path $r^{3}$ , as shown in Equation 46. But in the global path $r^{3}$ , $r_{1}^{3}$ and $r_{4}^{3}$ do not satisfy the continuity of direction, and ( 0→1 ) to ( 0→1 ) appear, so we need to change the direction of the remaining path $r_{4}^{3}$ , $r_{7}^{3}$ , $r_{10}^{3}$ , $r_{11}^{3}$ , $r_{12}^{3}$ and $r_{2}^{3}$ .


(46) $$\begin{array}{c} r^{3}=\{r_{1}^{3}\left(0\to 1\right),\;r_{4}^{3}\left(0\to 1\right),\;r_{7}^{3}\left(1\to 0\right),r_{10}^{3}\left(0\to 1\right),\\ r_{11}^{3}\left(1\to 0\right),r_{12}^{3}\left(0\to 1\right),r_{2}^{3}\left(1\to 0\right)\} \end{array}$$

where $r_{1}^{3}=\left\{A_{1},p_{322},B_{1}\right\}$ , $r_{4}^{3}=\left\{A_{4},p_{317},B_{4}\right\}$ , $r_{7}^{3}=\left\{B_{7},p_{320},A_{7}\right\}$ , $r_{10}^{3}=\left\{A_{10},p_{324},p_{319},B_{10}\right\}$ , $r_{11}^{3}=\left\{B_{11},p_{321},A_{11}\right\}$ , $r_{12}^{3}=\left\{A_{12},p_{323},B_{12}\right\}$ , $r_{2}^{3}=\left\{B_{2},p_{318},A_{2}\right\}$

We change the direction of the local path by modifying the order of the points of the local path, as shown in Equation 47. The robot continues driving along the updated path $r^{3}$ . **Figure 9D** shows that at 522 seconds, robot $R_{3}$ moves to the last working path, and the black path in the figure clearly shows the updated new path.


(47) $$\begin{array}{c} {\text{r}}^{3}=\{{\text{r}}_{1}^{3}\left(0\to 1\right),{\text{r}}_{4}^{3}\left(1\to 0\right),{\text{r}}_{7}^{3}\left(0\to 1\right),{\text{r}}_{10}^{3}\left(1\to 0\right),\\ {\text{r}}_{11}^{3}\left(0\to 1\right),{\text{r}}_{12}^{3}\left(1\to 0\right),{\text{r}}_{2}^{3}\left(0\to 1\right)\} \end{array}$$

where $r_{1}^{3}=\left\{A_{1},p_{322},B_{1}\right\}$ , $r_{4}^{3}=\left\{B_{4},p_{317},A_{4}\right\}$ , $r_{7}^{3}=\left\{A_{7},p_{320},B_{7}\right\}$ , $r_{10}^{3}=\left\{B_{10},p_{319},p_{324},A_{10}\right\}$ , $r_{11}^{3}=\left\{A_{11},p_{321},B_{11}\right\}$ , $r_{12}^{3}=\left\{B_{12},p_{323},A_{12}\right\}$ , $r_{2}^{3}=\left\{A_{2},p_{318},B_{2}\right\}$ .

**Figure 10:**
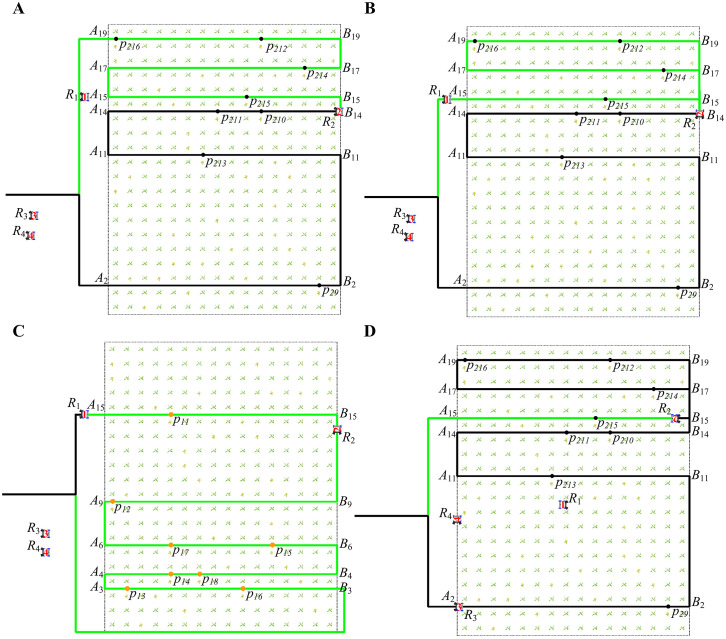
The paths of robots moving in different directions on the same working path at different times (the second case). **(A)** The path of robot $R_{2}$ at 10 seconds. **(B)** The path of robot $R_{2}$ at 13 seconds. **(C)** The path of robot $R_{1}$ at 13 seconds. **(D)** The path of robot $R_{2}$ at 173 seconds.

**Figure 10** shows the second case. Robots $R_{1}$ and $R_{2}$ move to the same working path $A_{15}B_{15}$ from other paths. **Figure 10A** shows that at 10 seconds, robot $R_{2}$ is about to leave its current working path $A_{14}B_{14}$ and move to the next working path $A_{15}B_{15}$ . **Figure 10B** shows that at 13 seconds, robot $R_{2}$ reaches the transition path $B_{14}B_{15}$ and begins to move toward the working path $A_{15}B_{15}$ . At this time, it is detected that another robot $R_{1}$ (**Figure 10C** shows the operational status of $R_{1}$ at 13 seconds) is also moving toward the working path $A_{15}B_{15}$ according to the itinerary table.

Since $R_{1}$ has a higher priority than $R_{2}$ , in order to avoid collision, $R_{2}$ abandons the current local path $r_{15}^{2}$ and re-plans a new path. The global path update method of $r^{2}$ is the same as Equations 46, 47. The updated path $r^{2}$ of $R_{2}$ is as shown in Equation 48. **Figure 10D** shows that at 173 seconds, the robot $R_{2}$ moves to the last working path, and the black path in the figure clearly shows the updated new path.


(48) $${\text{r}}^{2}=\left\{{\text{r}}_{2}^{2}\left(0\to 1\right),{\text{r}}_{11}^{2}\left(1\to 0\right),{\text{r}}_{14}^{2}\left(0\to 1\right),{\text{r}}_{17}^{2}\left(1\to 0\right),{\text{r}}_{19}^{2}\left(0\to 1\right),{\text{r}}_{15}^{2}\left(1\to 0\right)\right\}$$

where $r_{2}^{2}=\left\{A_{2},p_{29},B_{2}\right\}$ , $r_{11}^{2}=\left\{B_{11},p_{213},A_{11}\right\}$ , $r_{14}^{2}=\left\{A_{14},p_{211},p_{210},B_{14}\right\}$ , $r_{17}^{2}=\left\{B_{17},p_{214},A_{17}\right\}$ , $r_{19}^{2}=\left\{A_{19},p_{216},p_{212},B_{19}\right\}$ , $r_{15}^{2}=\left\{B_{15},p_{215},A_{15}\right\}$ .

#### 4.4.2 Working path and same movement direction

When the robots have the same serial number $j_{c}$ and the same moving direction, there are two cases: The robots maintain the current speed and move in the order of one after the other. The second is that when one of the robots stops working at the target point, the other robots at the back must wait for the front robot to complete the work.

**Figure 11:**
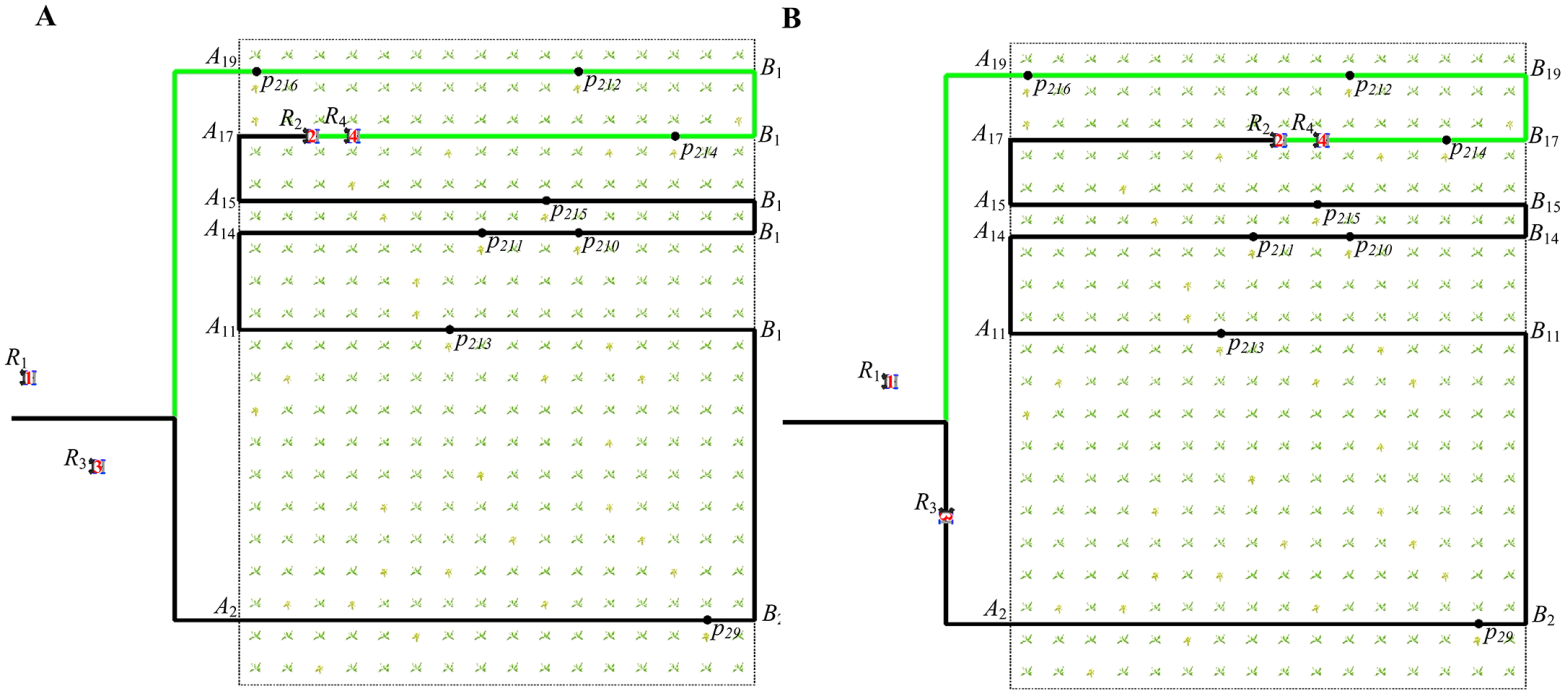
The paths of robots moving in the same direction on the same working path at different times (the first case). **(A)** The path of robot $R_{2}$ at 11 seconds. **(B)** The path of robot $R_{2}$ at 31 seconds.

**Figure 11** shows the first case. Robots $R_{2}$ and $R_{4}$ maintain a certain speed and distance, and move in a front-to-back sequence. **Figure 11A** shows the states of robots $R_{2}$ and $R_{4}$ at 11 seconds. **Figure 11B** shows that at 31 seconds, robot $R_{2}$ and $R_{4}$ keep moving forward in this state.

**Figure 12:**
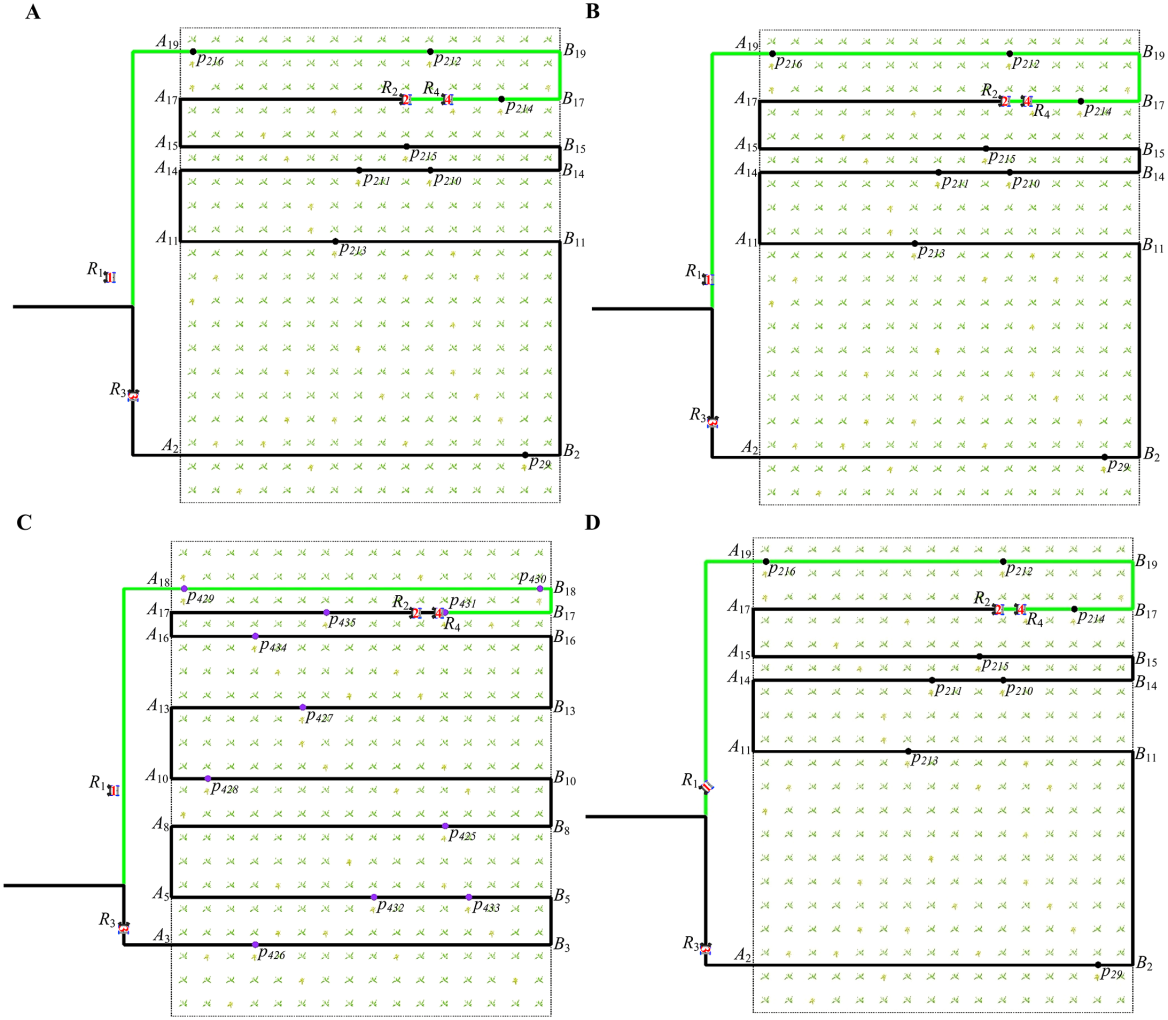
The paths of robots moving in the same direction on the same working path at different times (the second case). **(A)** The path of robot $R_{2}$ at 33 seconds. **(B)** The path of robot $R_{2}$ at 36 seconds. **(C)** The path of robot $R_{4}$ at 36 seconds. **(D)** The path of robot $R_{2}$ at 39 seconds.

**Figure 12** shows the second case. Robot $R_{4}$ stops moving and performs the spraying operation after reaching the target point $p_{431}$ . Robot $R_{2}$ stops moving, waiting for robot $R_{4}$ to complete the operation. **Figure 12A** shows that at 33 seconds, robot $R_{2}$ moves toward $R_{4}$ along the path. As depicted in **Figure 12B**, robot $R_{2}$ detects the halt of $R_{4}$ at 36 seconds, and $R_{2}$ maintains a safe distance from $R_{4}$ when it stops. **Figure 12C** shows that at 36 seconds, robot fpls.2024.1393541 stops at the target point $p_{431}$ . **Figure 12D** shows that at 39 seconds, robot $R_{2}$ stops moving until $R_{4}$ completes the work.

#### 4.4.3 Transition path and same movement direction

When the robot is on the transition path and moves in the same direction as other robots, there are two cases: One is that the robot keeps the current speed and follows the others. The other is that when one of the robots turns to the working path, the robots behind stop at a safe distance and wait for the turn to finish.

The first case is the same as the first case in 4.4.2. As long as the safe distance between robots is ensured, no other operations are performed.

**Figure 13:**
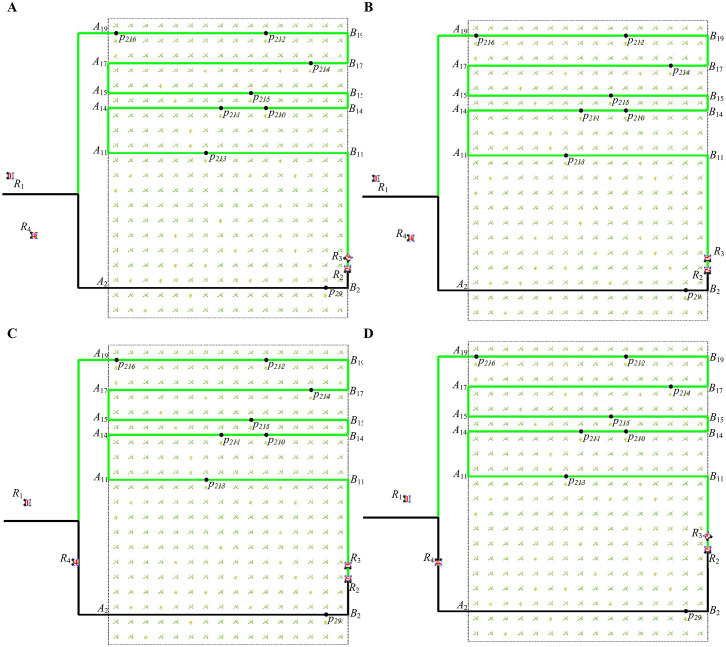
The paths of robots moving in the same direction on different working paths at different times. **(A)** The path of robot $R_{2}$ at 11   seconds. **(B)** The path of robot $R_{2}$ at 15 seconds. **(C)** The path of robot $R_{1}$ at 22 seconds. **(D)** The path of robot $R_{2}$ at 30 seconds.

**Figure 13** shows the second case, robots $R_{2}$ and $R_{3}$ are moving on the transition path. **Figure 13A** shows that at 11 seconds, robot $R_{2}$ detects that $R_{3}$ is moving from the working path to the transition path $B_{2}B_{11}$ , and $R_{2}$ stops moving and waits for $R_{3}$ to complete the turn. **Figure 13B** shows that at 15 seconds, after $R_{3}$ completes its turn, $R_{2}$ and $R_{3}$ maintain a certain safe distance and move along the transition path $B_{2}B_{11}$ . **Figure 13C** shows that at 22 seconds, robot $R_{2}$ maintains a safe distance from $R_{3}$ and moves in the same direction. **Figure 13D** shows that at 30 seconds, robot $R_{3}$ switches from the transition path $B_{2}B_{11}$ to the working path $A_{7}B_{7}$ , and robot $R_{2}$ stops at a safe distance, waiting for robot $R_{3}$ to finish the turn.

#### 4.4.4 Transition path and different movement directions

**Figure 14:**
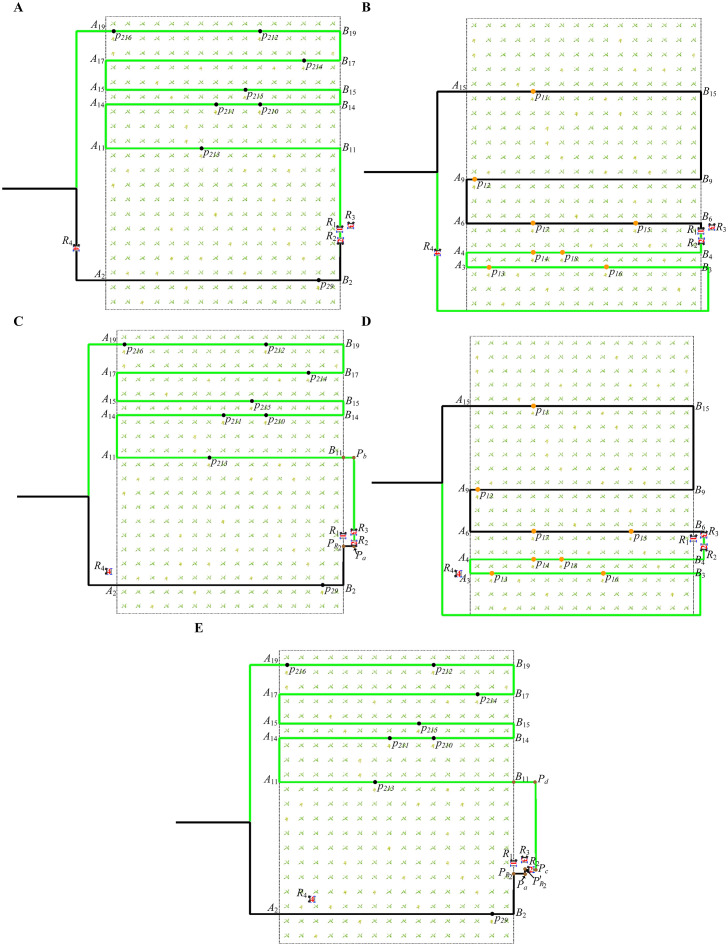
The paths of robots moving in different directions on different working paths at different times. **(A)** The path of robot $R_{2}$ at 31   seconds. **(B)** The path of robot $R_{1}$ at 31 seconds. **(C)** The path of robot $R_{2}$ at 47 seconds. **(D)** The path of robot $R_{3}$ at 47 seconds. **(E)** The path of robot $R_{2}$ at 56 seconds.

**Figure 14** shows that robots $R_{1}$ , $R_{2}$ , and $R_{3}$ are moving on the transition path. The $j_{c}$ and $j_{l}$ of $R_{1}$ are 4 and 6 respectively. The $j_{c}$ and $j_{l}$ of $R_{2}$ are 11 and 2 respectively. The $j_{c}$ and $j_{l}$ of $R_{3}$ are 4 and 6 respectively. According to Equation 6, their directions on the transition path are down, up, and down respectively. The moving direction of $R_{2}$ is different from that of $R_{1}$ and $R_{3}$ . Change the priority of $R_{1}$ , $R_{2}$ , and $R_{3}$ to 0, 2, and 1, respectively.

**Figure 14A** shows that at 31 seconds, robot $R_{2}$ detects that $R_{1}$ (**Figure 14B** shows the global path of $R_{1}$ at 31 seconds) is approaching from the opposite direction. Since the priority of $R_{2}$ is lower than that of $R_{1}$ , $R_{2}$ plans the obstacle avoidance path according to Section 3.4.2. $R_{2}$ translates the current local path a certain distance to the right and adds a new obstacle avoidance path $r_{t}^{1}$ , as shown in Equation 49.


(49) $$r_{t}^{1}=\left\{P_{R_{2}},P_{a},P_{b},B_{11}\right\}$$

Then, we directly insert $r_{t}^{1}$ between $r_{2}^{2}$ and $r_{11}^{2}$ in $r^{2}$ , as shown in Equation 50. $R_{2}$ moves according to updated $r^{2}$ . **Figure 14C** shows the new path of $R_{2}$ .


(50) $$r^{2}=\left\{r_{2}^{2}\left(0\to 1\right),r_{t}^{1},r_{11}^{2}\left(1\to 0\right),r_{14}^{2}\left(0\to 1\right),r_{17}^{2}\left(1\to 0\right),r_{19}^{2}\left(0\to 1\right),r_{15}^{2}\left(1\to 0\right)\right\}$$

where $r_{2}^{2}=\left\{A_{2},p_{29},B_{2}\right\}$ , $r_{t}^{1}=\left\{P_{R_{2}},P_{a},P_{b},B_{11}\right\}$ , $r_{11}^{2}=\left\{B_{11},p_{213},A_{11}\right\}$ , $r_{14}^{2}=\left\{A_{14},p_{211},p_{210},B_{14}\right\}$ , $r_{15}^{2}=\left\{B_{15},p_{215},A_{15}\right\}$ , $r_{17}^{2}=\left\{A_{17},p_{214},B_{17}\right\}$ , $r_{19}^{2}=\left\{B_{19},p_{212},p_{216},A_{19}\right\}$ .

$R_{2}$ detects a new robot $R_{3}$ (**Figure 14D** shows the global path of $R_{3}$ at 47 seconds) approaching from the opposite direction at 47 seconds in **Figure 14C**. Since $R_{2}$ has a lower priority than $R_{3}$ , $R_{2}$ plans an obstacle avoidance path to avoid colliding with $R_{3}$ . Just like the rules for avoiding $R_{1}$ , move the current local path $r_{t}^{1}$ a certain distance to the right, and then update the local path $r_{t}^{1}$ , as shown in Equation 51.


(51) $$r_{t}^{1}=\left\{P_{R_{2}},P_{a},P_{R_{2}}^{\prime },P_{c},P_{d},B_{11}\right\}$$

Then, we directly insert the updated $r_{t}^{1}$ between $r_{2}^{2}$ and $r_{11}^{2}$ in $r^{2}$ , as shown in Equation 52. $R_{2}$ moves according to updated $r^{2}$ . **Figure 14E** shows the new path of $R_{2}$ at 56 seconds.


(52) $$r^{2}=\left\{r_{2}^{2}\left(0\to 1\right),r_{t}^{1},r_{11}^{2}\left(1\to 0\right),r_{14}^{2}\left(0\to 1\right),r_{17}^{2}\left(1\to 0\right),r_{19}^{2}\left(0\to 1\right),r_{15}^{2}\left(1\to 0\right)\right\}$$

where $\;r_{t}^{1}=\left\{P_{R_{2}},P_{a},P_{R_{2}}^{'},P_{c},P_{d},B_{11}\right\}$ .

### 4.5 Analysis of experimental results

#### 4.5.1 Analysis of multi-robot collision avoidance results

We recorded the number of times the multi-robot system avoided collisions and the time spent executing tasks for various numbers of target points, as shown in **Table 2**. We randomly generated sets of 15, 25, 35, and 45 target points and conducted 10 experiments for each set, with target points randomly generated in each experiment. For the multi-robot system, we recorded the total number of collisions avoided and the total time spent on task execution for each robot. For a single robot, we recorded only the total time spent on the spraying task.

**Table 2:** Experimental results (The total number of collisions avoided and the total time spent on the task for each robot).

Number of target points		15		25		35		45	
System	Robot	Time (s)	Number of collisions avoided	Time (s)	Number of collisions avoided	Time (s)	Number of collisions avoided	Time (s)	Number of collisions avoided
Multi-robot system	*R_1_*	2562	27	3617	32	4473	37	4887	32
	*R_2_*	2575	33	3282	42	4242	44	4972	40
	*R_3_*	2782	35	3619	25	4468	29	5353	42
	*R_4_*	2770	24	3509	31	4157	30	5157	31
Single robot system		6264	\	8589	\	8765	\	9024	\

As shown in **Table 2**, under different numbers of target points, the time taken by the multi-robot system to complete the task is much shorter than that of a single robot. Specifically, for 15 target points, the multi-robot system avoids collisions 119 times in total, saving 55.6% of the time compared to a single robot. For 25 target points, the multi-robot system avoids collisions 130 times in total, saving 57.9% of the time compared to a single robot. For 35 target points, the multi-robot system avoids collisions 140 times in total, saving 48.9% of the time compared to a single robot. For 45 target points, the multi-robot system avoids collisions 145 times in total, saving 40.7% of the time compared to a single robot. The experimental results show that under the same number of target points, the multi-robot system can complete the spraying task while avoiding collisions or conflicts between robots, and the completion time can be reduced by more than 40%.

#### 4.5.2 Comparison experiment with other methods

To evaluate our method, we compare it with the classic Reciprocal Velocity Obstacles (RVO) (Van den Berg et al., 2008) and Optimal Reciprocal Collision Avoidance (ORCA) (Niu et al., 2021) algorithms. In terms of time complexity, the time complexity of RVO and ORCA algorithms is *O*(*n* ^2^). As shown in **Algorithms 1–3**, their time complexity are *O*(*n*), *O*(*n* ^2^), and *O*(*n*), respectively. Therefore, the overall time complexity of our algorithm is *O*(*n* ^2^).

Since the width of the working path in the field is relatively narrow, it cannot support two robots moving side by side. When two robots move toward each other on the working path (as shown in **Figure 2B**), if the low-priority robot performs an evasive action, it will inevitably collide with the crops. Therefore, we made some modifications to the RVO and ORCA algorithms. If they are detected to be moving in opposite directions on the working path, the low-priority robot stops and waits for the other robot to complete the work before continuing the work. Under the same settings, ten spraying tests with the same number and the same order are conducted for each collision avoidance method with different numbers of target points. The completion time mainly reflects the efficiency of different methods, and the number of collisions avoided reflects the performance from the side.

The experimental results are shown in **Table 3**, demonstrating that the proposed collision avoidance algorithm outperforms the RVO and ORCA algorithms. Specifically, for 15 target points, the completion time of our algorithm is reduced by 5.2% and 2.9% compared with RVO and ORCA, respectively. This is because when the number of target points is relatively small, the probability of collision or conflict between robots in a large field is relatively small, so the total completion time is not much different. For 25 target points, the completion time is reduced by 9.8% and 3.8% compared with the RVO and ORCA algorithms, respectively. For 35 target points, the completion time is reduced by 14.9% and 8.2% compared with the RVO and ORCA algorithms, respectively. For 45 target points, the completion time is reduced by 16.8% and 9.5% compared with the RVO and ORCA algorithms, respectively. As the number of target points increases, the likelihood of collisions or conflicts between robots also increases. Our proposed algorithm demonstrates better performance, with a greater reduction in completion time compared to RVO and ORCA. In addition, the number of collision avoidance of our proposed algorithm is less than that of RVO and ORCA. This is because our collision avoidance algorithm can pre-judge potential collisions or conflicts on the working path, thereby reducing their occurrence. In summary, our proposed algorithm can complete the spraying task on large-scale fields in a timely and efficient manner, exhibiting high operational efficiency.

**Table 3:** Experimental results of different collision avoidance methods.

Number of target points	15		25		35		45	
Method	Time (s)	Number of collisions avoided	Time (s)	Number of collisions avoided	Time (s)	Number of collisions avoided	Time (s)	Number of collisions avoided
RVO	2935	142	4012	158	5255	192	6432	211
ORCA	2864	140	3761	152	4872	180	5917	185
Ours	2782	119	3619	130	4473	140	5353	145

### 4.6 Summary

We designed a simulation environment for precise spraying of sweet potato fields, and then experimentally verified the collision avoidance method we proposed against four types of collisions or conflicts that may occur between multiple robots in the field. We demonstrate that our multi-robot collision avoidance method can be well deployed on robots even though they only have communication modules, positioning modules and low-cost control chips. We validate the collision avoidance strategy in various collision or conflict scenarios and show that our method can run robustly for long periods of time without collisions.

## 5 Conclusion

In this article, we propose a multi-robot collision avoidance method that only uses point-line maps and real-time robot positions to determine the relationship between robots, make reasonable decisions, and avoid collisions between robots. We evaluate the performance of our method through a series of comprehensive experiments and demonstrate that the collision avoidance method is simple and efficient in terms of success rate, safety, and navigation efficiency. For the current situation where only a single robot is used to complete the operation, the method we proposed can greatly improve the efficiency of farmland robot operations. At the same time, the method we proposed has extremely low requirements for robot hardware performance, which greatly reduces the cost of each robot, thereby reducing farmers’ farmland management costs and labor intensity, and indirectly increasing farmers’ economic income. It provides the theoretical basis and technical support for reducing the cost of multi-robot systems and accelerating the promotion and application of multi-robot systems in agriculture.

Our work is a first step toward reducing robot costs, avoiding robot collisions, and improving the efficiency of multi-agricultural robots. Although we are fully aware that as a local collision avoidance method, our approach cannot completely replace reinforcement learning-based multi-robot path planners when scheduling. Our future work will be how to extend our method to larger-scale robot systems at low cost and apply this method to real field environments to achieve a safe and efficient multi-robot collision avoidance method.

## Data Availability

The original contributions presented in the study are included in the article/supplementary material. Further inquiries can be directed to the corresponding author.
